# Redefining awn development in rice through the breeding history of Japanese awn reduction

**DOI:** 10.3389/fpls.2024.1370956

**Published:** 2024-05-16

**Authors:** Mao Suganami, Hideki Yoshida, Shinya Yoshida, Mayuko Kawamura, Eriko Koketsu, Makoto Matsuoka, Soichi Kojima

**Affiliations:** ^1^Faculty of Food and Agricultural Sciences, Institute of Fermentation Sciences, Fukushima University, Fukushima, Japan; ^2^Hyogo Prefectural Research Center for Agriculture, Forestry and Fisheries, Kasai, Japan; ^3^Research Institute for Food and Agriculture, Ryukoku University, Ootsu, Japan; ^4^Bioscience and Biotechnology Center, Nagoya University, Nagoya, Japan; ^5^Graduate School of Agricultural Science, Tohoku University, Sendai, Japan

**Keywords:** *An-1*, awn, breeding history, cold resistance, domestication, *EPFL1*, GWAS, rice

## Abstract

The study challenges the conventional understanding of awn loss as a domestication syndrome, showing instead that many awned varieties continued to be widely grown in Japan until the early twentieth century and that selection for awn reduction was active at that time, demonstrating that awn loss is not a domestication syndrome but “a trait that emerged during crop improvement”. Although selection for awnless mutants was carried out independently using different types of awned cultivars in the early twentieth century in Japan, awn loss was caused by the mutation in *OsEPFL1.* This suggests that a single mutant haplotype of *OsEPFL1* was conserved in the genomes of different cultivars and subsequently selected within each line to meet the demand for awnless varieties. The study also conducts phylogenetic analyses of *EPFL1* in 48 grass plants, revealing its unique involvement in awn formation in rice while potentially playing a different role in the domestication of other grass plants. Finally, an attempt is made to isolate an awn-forming gene that has not been identified from the awned rice cultivar “Omachi”, which is still cultivated in Japan. The results presented in this paper provide a new perspective on domestication against the conventional understanding of awn development, shedding light on its potential as a useful organ for breeding to mitigate environmental stress.

## 1 Introduction

The awn is a bristle-like organ found at the tips of the lemmas of some grass species, such as rice, wheat, and barley, and has been thought to prevent animals from eating the seeds and also to help disperse the seeds. In human rice cultivation, however, eliminating the awn has been discussed as having several advantages. First, awns can affect harvesting efficiency by influencing seed behavior during harvesting, processing efficiency during postharvest operations such as cleaning, sorting, and milling, and potentially the storage and transportation of harvested crops (Takahashi et al., 1986a). In addition, some papers have reported that the development of rice awns has a negative impact on yield (Luo et al., 2013; Gu et al., 2015; Jin et al., 2016), as no chlorenchyma was observed in rice awns, in contrast to wheat or barley (Toriba et al., 2010). Indeed, wild rice has retained long awns for successful seed dispersal, but almost all modern cultivars exhibit the awnless phenotype (Furuta et al., 2015). Consequently, the elimination of awns has been considered one of the key events in the process of plant domestication and cultivation (Hua et al., 2015; Amarasinghe et al., 2020; Luong et al., 2022).

On the other hand, Svizzero et al. (2019) argued that a long awn is advantageous for humans, not only because it prevents seed predation by animals but also because it increases the probability that the awn will keep grains on the panicle even if grains are shed from the panicle before the shattering habit is eliminated, and that the awn is a trait that increases yield potential in the absence of advanced cultivation. Based on this argument, they concluded that awn reduction was not a domestication syndrome. Furthermore, Kato (1911) studied the situation of rice cultivation in Japan in the early twentieth century and pointed out that many varieties grown at high latitudes were awned, probably because awning can increase cold tolerance (see below). These arguments suggest that the loss of awns is not a domestication syndrome but at best a trait for crop improvement or perhaps a useful organ for breeding to alleviate environmental stress.

In this paper, we investigated the molecular mechanism based on awn reduction breeding, which was actively carried out in Japan in the early twentieth century. Many documents state that this awn-reducing breeding was done independently on different awned varieties, but our examination of the genomes of these newly obtained awnless varieties revealed that they were all caused by mutations in a known awn-forming gene, epidermal patterning factor-like protein 1 (*OsEPFL1*, Takata et al., 2013). Furthermore, this *EPFL1* gene is conserved in many plant species of the Poaceae but has curiously disappeared from highly domesticated plant groups such as wheat, barley, rye, maize, and sorghum. Moreover, the relationship between this gene and awn formation is restricted to rice and is not associated with awn formation in other plant species. Finally, we have attempted to isolate the awn-forming gene that has not been identified in “Omachi”, which is still cultivated in Japan, and discussed its function.

## 2 Materials and methods

### 2.1 Plant materials and genotyping

Rice varieties used in this study were obtained from NARO Genebank (https://www.gene.affrc.go.jp/databases-plant_search_char.php?type=428), the Kyushu University Rice Database (https://shigen.nig.ac.jp/rice/rice-kyushu/htdocs/main.html) or purchased directly from the seed suppliers. DNA preparation and genotyping were performed as previously described (Yano et al., 2016; Suganami et al., 2023), with slight modifications. DNA for genotyping was isolated from leaves using a DNeasy Plant Mini Kit (Qiagen, Hilden, Germany) and fragmented into approximately 500 bp using Covaris S2 (Covaris, Brighton, UK). The NEBNext DNA Library Prep Reagent Set (New England Biolabs, Ipswich. MA) was used for DNA library construction. Paired-end sequencing was performed using the Illumina Hiseq system (Illumina Co. Ltd., San Diego, CA) with a read length of 100–150 bp. All reads were mapped against Os-NipponbareReference-IRGSP-1.0 pseudomolecules (all.con ver.7, Kawahara et al., 2013), and fastq files were converted into samfiles using the bwa-mem command of BWA software ver0.7.18 (Li, 2013). Commands samtools-view, samtools-sort, and samtools-index of Samtools software ver1.9 (Li et al., 2009) were used to generate, sort, and index bam files successively. The variants for each accession were called using the GATK HaplotypeCaller (release 4.1.9.0) with the “.g.vcf” extension (McKenna et al., 2010). GATK CombineGVCFs was used for joint genotyping to produce a single VCF file for each compared pair and group. Homozygous polymorphisms in all the compared genomes were used for the prediction of causative polymorphisms.

### 2.2 Phylogenetic tree analysis

For the phylogenetic tree of the genome of Japanese temperate rice varieties, the SNPhylo pipeline (Lee et al., 2014) was used to create a maximum likelihood phylogenetic tree based on representative genomic SNPs for temperate *japonica* varieties shown in **Supplementary Table S2**. The pipeline was employed using default parameters and 100 bootstrap replicates to create the bootstrapped maximum likelihood tree. For phylogenetic tree of EPFL1/2 and ONAC085 protein, sequence alignment was performed using the ETE3 3.1.2 function “build” (Huerta-Cepas et al., 2016) as implemented by GenomeNet (https://www.genome.jp/tools/ete/), and phylogenetic tree creation was performed with the model PROTGAMMAJTT, and default parameters RAxML v8.2.11 were used for inference. Branch support was computed with 100 bootstraps.

### 2.3 Evaluation of awn development

“Awn development” in the 399 lines (Nagoya Panel) is scored by looking at the entire spikelet of the panicle. When awn formation occurs vigorously in the upper to middle part of the panicle with a long length and also some frequency of awn formation around the lower part, we categorized it as “vigorous”. If there were clear awns with some length at the tip, but such awn formation was apparently weakened, we categorized them as “moderate” and “hardly” if there were almost no awn or only a short one at the tip.

In addition, there were “degree of awn formation” and “awn length” in the 153 lines (Hyogo Panel) measured over 12 years from 1992 to 2003 as historical data. These are two phenotypic data scored by specifically looking at the spikelet at the tip of the panicle, according to the Rice Assesment Guidelines for Seed Registration (2024). This field was an environment in which Nipponbare consistently exhibited the awned phenotype, whereas Yamadanishiki did not have awn.

### 2.4 Transgenic analysis

Transgenic analysis was performed according to Yano et al. (2016) and Yoshida et al. (2022). A genomic DNA fragment of full-length *ONAC085* and *OsGEP* plus approximately 2,000 bp upstream and 500 bp downstream regions was PCR amplified from genomic DNA extracted from leaves of Japanese rice varieties, Nipponbare and Yamadanishiki. *ONAC085* genomic fragments were produced with the primer pair 5′-TCCCCCGGGCCTTCGGATTAGTGTTTATTC-3′ and 5′-TCCCCCGGGAAACTAAAATTTAGCTTGC-3′. *OsGEP* genomic fragments were produced with the primer pair 5′-GCGGATCCGAATGGTTTTGATAGTTAAG-3′ and 5′-CGGGATCCAACAAACCTCACAATAACAG-3′. The *ONAC085* and *OsGEP* fragments were subcloned into the *Sma*I or *Bam*HI sites of pCAMBIA1380 using the NEBuilder HiFi DNA Assembly master mix (New England BioLabs), respectively.

### 2.5 GWAS

We used the historical data of two phenotypic data degree of awn formation and awn length in the 153 lines (Hyogo Panel) for the GWAS. The population structure of the 153 varieties was estimated by PCA, which was performed using the R package “SNPRelate” version 4.2 (Zheng et al., 2012). A linear mixed model (LMM) was used for GWAS. GWAS was performed using the R package “rrBULP” version 4.3, according to Yano et al. (2016). SNPs with minor allele frequencies (5%) and missing rates (10%) were filtered out for the GWAS study. The kinship matrix was included in the calculation, but the principal component was not included as a fixed effect. The vcf data are available in zenodo at https://doi.org/10.5281/zenodo.10990900.

### 2.6 QTL mapping by bi-parent population

QTL analysis was performed according to Yoshida et al. (2023). The QTL analysis by the population crossed between Reiho and Yamadanishiki was performed with 91 doubled haploid lines (DHLs) produced by the anther culture method. Genotypes were determined using 276 genomewide DNA markers (SNPs, 167; SSRP, 68; RAPD, 34; and AFLP, 7) (Yoshida et al., 2002).

### 2.7 Measurement of transpiration and respiration of awn and grain

Aikoku, a variety with long awn, grown in a paddy field and greenhouse at Tohoku University (Miyagi, Japan), was used for the measurements. Measurements were taken during the morning hours of a sunny day just after ear emergence (2022/8/21). Using a Li-Cor LI-6800 portable gas exchange system, measurements were made with six to eight awns and spikelets clamped in a 3 × 3 chamber at a chamber temperature of 30°C, ambient CO_2_ of 40 Pa, and a photosynthetic photon flux density of 1,500 μmol photons m^−2^ s^−1^. Results are shown per an awn.

### 2.8 Database URLs

RAP-DB (https://rapdb.dna.affrc.go.jp/), Rice Genome Annotation Project (http://rice.uga.edu/), Ensembl Plants (https://plants.ensembl.org/), Phytozome (phytozome-next.jgi.doe.go), RiceXPro (https://ricexpro.dna.affrc.go.jp/), Transcriptome ENcyclopedia Of Rice (https://tenor.dna.affrc.go.jp/), and Rice SNP-Seek Database (https://snp-seek.irri.org/).

## 3 Result

### 3.1 The breeding of Japanese rice varieties for awn reduction was actively conducted at the beginning of the twentieth century

**Figure 1:**
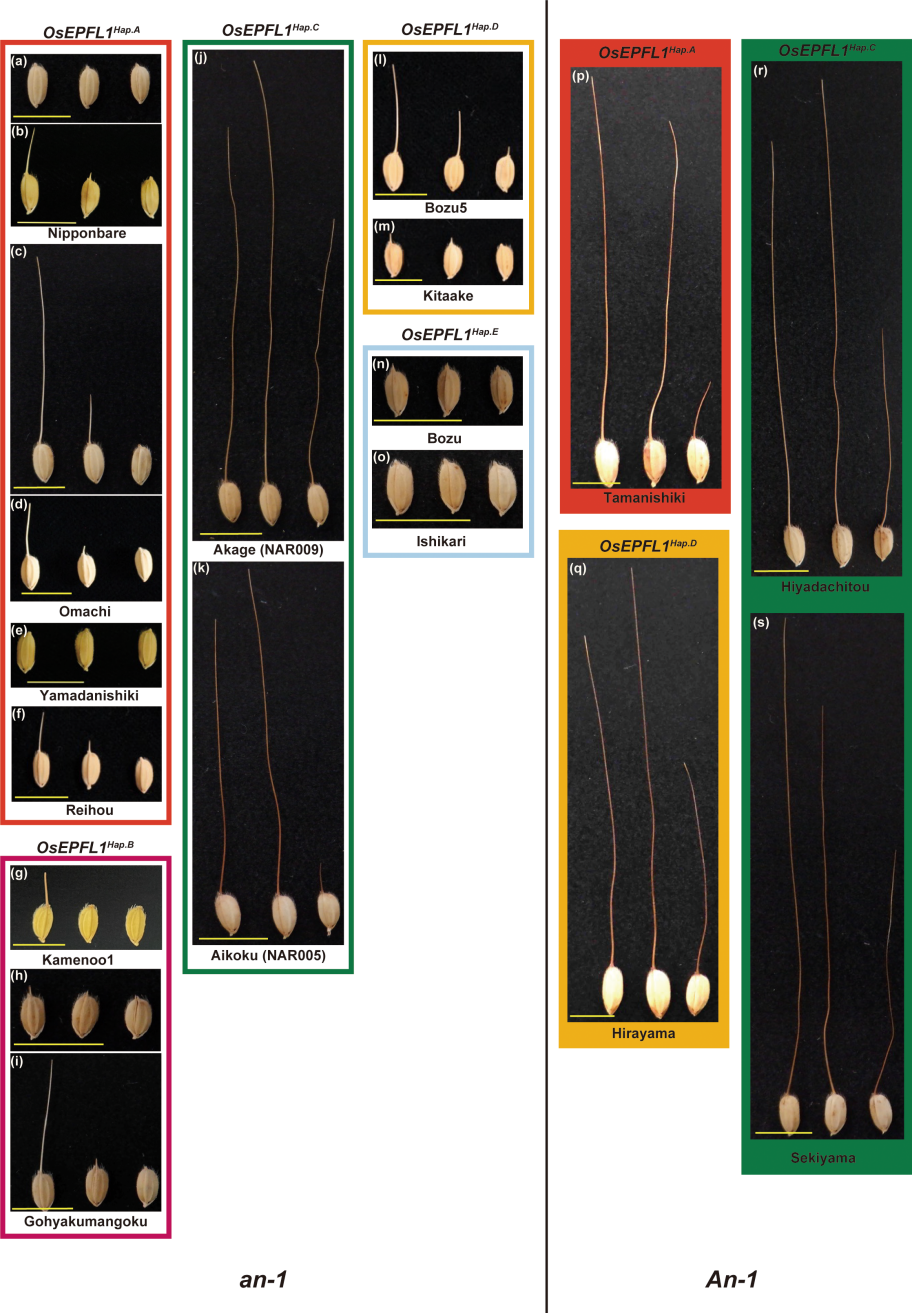
Awn morphology of Japanese rice varieties classified by *OsEPFL1* and *An-1*. The eight groups are classified according to the haplotypes of the two main awn formation genes, *OsEPFL1* and *An-1*, and a typical awn morphology was shown for each. Awn formation varies according to the position of the spikelets on the panicle, with spikelets at the top forming the longest awns (left), while those at the base have shorter awns (right), and their formation decreases from top to bottom (center shows at the middle). Under this situation, varieties carrying the functional *OsEPFL1* and/or *An-1* usually form awns of some length even in the basal part of the panicle, which are categorized as “vigorously” in **Supplementary Table S2** **(J**, **K**, **P–S)**. Some varieties, even lacking their functional genes, can also form awns of some length on the uppermost spikelets, but which are short or absent in the middle or lower spikelets **(C, I, L)**, which are categorized as “moderate”. Varieties that do not form awns with more than some length at the top of the panicle are categorized as “hardly” **(A, E–G, M–O)**. **(B**, **D**, **H)** showed a variation of awn development. Yellow bars indicate 1 cm.

**Table 1** is the first list of the top 10 rice varieties grown at the national level in Japan in the early twentieth century (Kato, 1908; Yasuda, 1955). Among these varieties, “Aikoku” and "Omachi" form the awn (**Figures 1D, K**), although the style of awn formation is different between the two (see below). Furthermore, according to documents, the awnless varieties in this list, such as “Shinriki”, “Ooba”, “Ishijiro”, “Kamenoo”, and “Bozu” were selected from awned varieties to awnless during the early twentieth century (**Supplementary Figure S1**: Nishio and Fujimaki, 2020). Thus, dominant rice varieties planted in the early twentieth century in Japan were either still developing awn, or even if some of them did not form awn, their one-generation progenitors could develop long awn. In fact, a list of rice varieties grown in the “Kaga domain”, located in the middle of Japan (Inao and Uchiyama, 1736), shows that 43% of the varieties had a long awn and 13% had a short awn, compared to 45% of the awnless varieties (**Supplementary Table S1**).

**Table 1:** Top 10 paddy rice cultivars in Japan in the early twentieth century.

Rank	1908			1925		
	Variety name	ID	Planted area (ha)	Variety name	ID	Planted area (ha)
1	Shinriki	JRC033	608	Shinriki	JRC033	410
2	Aikoku	JRC026	142	Aikoku	JRC026	162
3	Omachi	JRC032	141	Kamenoo	NAR029	158
4	Sekitori	NAR053	77	Bozu	NAR083	75
5	Takenari^a^	KOB050	74	Rikuu20	NAR105	65
6	Shiratama^a^	NAR049	64	Omachi	JRC032	61
7	Ooba	NAR022	59	Toyokuni^a^	None	60
8	Ishijiro	JAR023	51	Asahi^a^	JRC034	44
9	Miyako^a^	NAR099	45	Ooba	NAR022	34
10	Shirasasa^a^	None	32	Sekitori	NAR053	32

Yellow: cultivars with awn; orange; selected from the awned cultivars; gray: mixture of awned and awnless lines.

a No awn.

**Figure 2:**
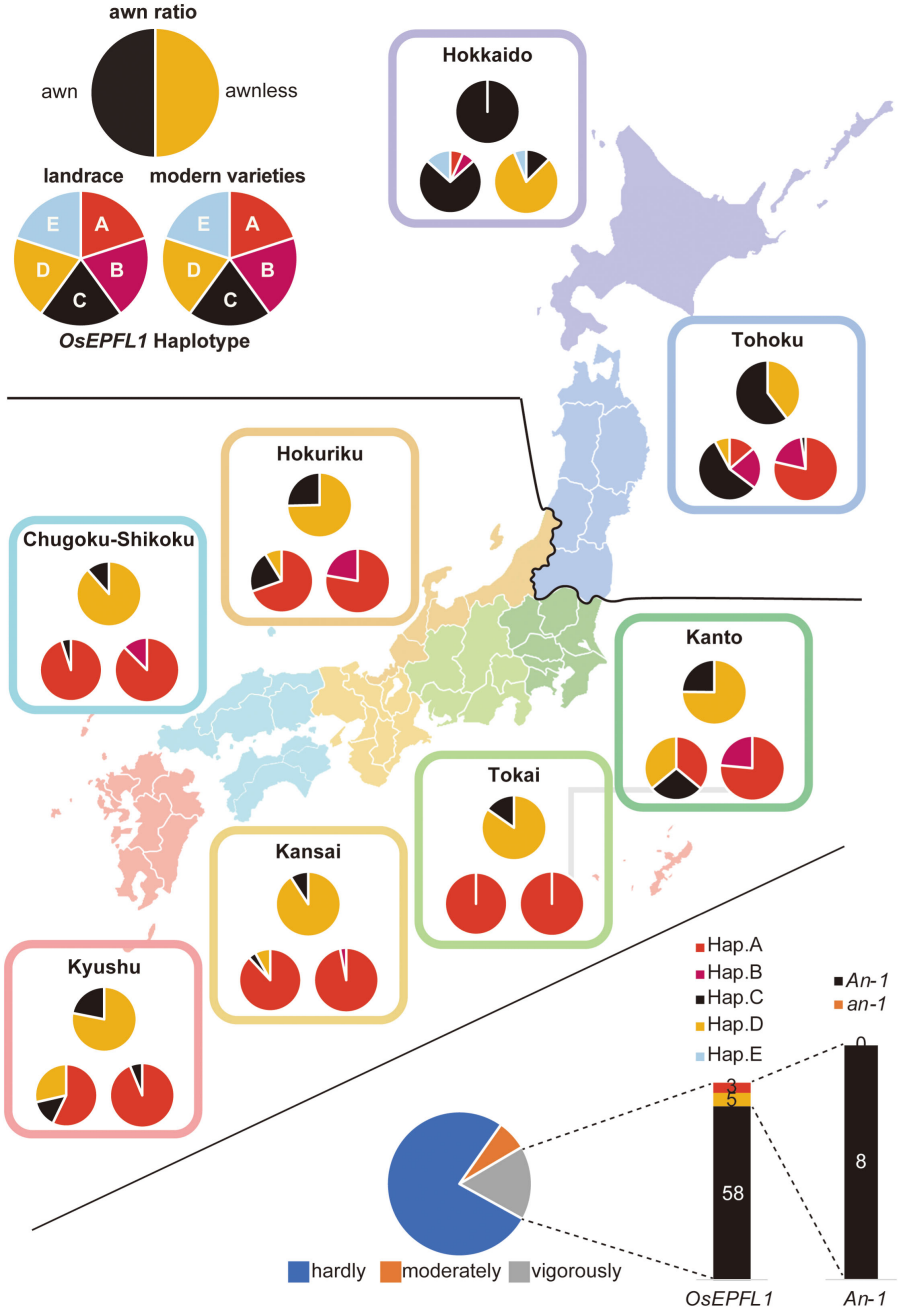
The cultivation of awned varieties was localized in the northern part of Japan. The localization of awned varieties was examined in three categories: the ratio of awned (black)/awnless (orange) based on a document on rice cultivation reported in Kato (1908), Kato (1911) (top pie chart), *OsEPFL1* haplotype of 190 landraces in our panel (bottom left), and 209 modern varieties (bottom right). Some landraces in Kanto and Kyushu have *OsEPFL1^Hap.D^* lines due to the abundance of upland rice among them, whereas many of the modern varieties in Hokkaido have *OsEPFL1^Hap.D^*.

Kato (1911) reported that, of the 38 representative cultivars grown in Japan at the beginning of the twentieth century, 13 were awned cultivars that were abundant in the high-latitude regions (**Figure 2A**). Thus, we focused on *OsEPFL1*, which we previously isolated as the sole gene involved in awn formation in Japanese cultivars (Yano et al., 2016; Suganami et al., 2023), and examined the regional distribution of its haplotypes (**Figure 2A**). Among landraces, we found five different haplotypes (**Supplementary Figure S2A**). The functional haplotype, *OsEPFL1Hap.C*, which can produce a long awn, tended to be localized in high-latitude regions such as Tohoku and Hokkaido, while most varieties grown in mid- and low latitudes had its defected haplotype, *OsEPFL1Hap.A* (**Figure 2A**).

As for why there are many awned varieties in the northern part of Japan, it was speculated that it may reduce the risk of nonmaturation of grains by compensating for the outflow of water that occurs during the maturation process by transpiration from awn, since low-temperature and high-humidity conditions suppress transpiration activity (Kato, 1911; Takahashi et al., 1986a, 1986b, Alterfa et al., 1986). Thus, we directly measured gas exchanges of Aikoku awns immediately after flowering and observed transpiration rates of 106~183 µmol s^−1^ per awn in the field (**Table 2**). Since transpiration from spikelets is about 400 µmol s^−1^ per spikelet, the transpiration rate of the awn contributes ~30% of that of the grain. We also observed awn transpiration in the greenhouse, but its level was almost one-third of that in the field, probably because the humidity in the greenhouse is much higher than in the field. On the other hand, since the gas exchange of CO_2_ was below the accuracy guarantee limit (0.1 μmol mol^−1^) in both measurements, both respiration and photosynthesis were hardly observed. This suggests that the rice awn does not contribute to grain development by respiration or photosynthesis but that its transpiration activity functions and contributes to grain development, especially under low-temperature conditions, as speculated by Kato (1911) (see Discussion).

**Table 2:** Transpiration and respiration of awn and grain for “Aikoku”.

			CO_2__s (µmol mol^−^¹)	CO_2__r (µmol mol^-1^)	H_2_O_s (mmol mol^-1^)	H_2_O_r (mmol mol^-1^)	Transpiration rate (µmol s^-1^)	Respiration rate (µmol s^-1^)
Field	Awn	Sample 1	400.04	399.98	28.17	27.89	182.91	0.11
			400.03	399.96	28.15	27.88	177.61	0.11
			400.01	399.97	28.13	27.87	167.36	0.09
		Sample 2	399.99	400.00	28.29	28.12	106.68	0.04
			399.99	399.99	28.29	28.12	106.10	0.04
			400.02	399.99	28.28	28.12	105.66	0.06
		Sample 3	399.95	399.98	27.73	27.53	129.69	0.04
			399.96	399.98	27.73	27.53	129.54	0.04
			399.95	399.99	27.72	27.53	123.02	0.03
	Grain	Sample 1	399.71	400.00	27.74	27.27	402.16	0.08
			399.70	400.00	27.74	27.28	399.95	0.09
			399.70	400.00	27.74	27.28	398.06	0.09
Greenhouse	Awn	Sample 1	399.97	399.98	28.68	28.63	34.80	0.01
			399.96	399.96	28.68	28.63	35.87	0.02
			399.97	400.02	28.68	28.62	36.99	−0.02
		Sample 2	400.03	399.99	28.84	28.80	41.56	0.06
			400.04	399.99	28.84	28.80	42.32	0.06
			400.03	399.98	28.84	28.80	42.82	0.06

Measurements were taken three times per sample.

### 3.2 OsEPFL1 dominantly regulates the awnless event in Japanese varieties

There are many documents describing that the selection of awnless varieties from awned ones was independently carried out in various places in Japan (e.g., **Supplementary Figure S1**: Nishio and Fujimaki, 2020). By comparing the genomes of the selected awnless and their parental awned varieties, we thought we could comprehensively isolate the genes involved in awn development and examine the relationship between *OsEPFL1* and “awned or not awned” in 399 lines (**Supplementary Table S2**). Because varieties from the early twentieth century or earlier, before the establishment of modern breeding, often had different genome structures even if they had the same name, we treated them as different strains with the same name by assigning an accession number to the variety name. Most of the long-awned varieties have the functional haplotype, *OsEPFL1^Hap.C^*, whereas Shinriki, Ishijiro, and Ooba in **Supplementary Figure S1**, which were selected independently from different awned parents according to documents, have the same defective haplotype, *OsEPFL1^Hap.A^*. Since *OsEPFL1^Hap.A^* is also found in many temperate and tropical *japonica* and also *aus* in the outside of Japan (**Supplementary Figure 2B**), it is very likely that this mutation occurred overseas, was introduced to Japan, and was then maintained in the populations of various varieties (since this mutation is recessive, a certain number of individuals in the population must exhibit the “awnless” trait). Under such a situation, the “awnless selection” might have occurred as if it were independent events within each breeding variety.

As mentioned, most of the Japanese awnless varieties have achieved their awn reduction by the *OsEPFL1^Hap.A^* mutation, but we also found other *OsEPFL1* mutations in some awnless varieties. *OsEPFL1^Hap.B^* is one of the other haplotypes with a 6-bp deletion of the cysteine residue coding region essential for OsEPFL1 activity (**Supplementary Figure S2A**). In total, 32 varieties, including Kamenoo, carry it (**Supplementary Table S2**). Among them, 21 varieties can be traced back to Kamenoo (**Supplementary Figure S3**), and we speculated that the origin of *OsEPFL1^Hap.B^* is this variety. According to a document (Nishio and Fujimaki, 2020), Kamenoo was selected in 1893 from a cold-tolerant variety, “Hiyadachitou”. We compared the whole genome structures of Kamenoo and Hiyadachitou by a phylogenetic tree analysis, whereas as there are five accessions of Kamenoo in NARO Genebank, we analyzed all of them. Of these five Kamenoo lines, NAR029 and NAR030 are closely related to each other, and NAR033 is also related to these two lines to some extent, but the other two lines (NAR032 and NAR007) are significantly different from the above three lines (**Supplementary Figures S4A, B**), indicating that the variety Kamenoo should be considered a heterogeneous population with large genome diversity. Furthermore, genomic homology between all accessions of Kamenoo and Hiyadachitou was also low, demonstrating that Kamenoo is not a genetic progeny of Hiyadachitou (**Supplementary Figure S4A**). On the other hand, the whole genome structure of three varieties, such as “Kamenoo1 (KYU022)”, “Kamenoo4 (KOB013)”, and “Kamenoo_Daikoku (NAR034)”, all of which have *OsEPFL1^Hap.B^*, showed almost the same as “Kamenoo (NAR029)” (**Supplementary Figure S4B**), demonstrating that these varieties are selected and established as progenies from “a specific Kamenoo individual (NAR029)”, as described by breeding reports (Nishio and Fujimaki, 2020).

To investigate the origin of *OsEPFL1^Hap.B^*, we used RiceVarMap v2.0 (https://ricevarmap.ncpgr.cn/) to determine whether varieties with this mutation exist abroad and found 12 ones (**Supplementary Table S3**). Of these, five are of Japanese origin, one is of unknown origin, and four cannot be distinguished between modern and landrace. However, the remaining two varieties, “Baxang” from Vietnam and “Arborio” from Italy, are landraces that have been grown for a long time in these different places (**Supplementary Table S3**). It is difficult to imagine that Japanese varieties carrying *OsEPFL1^Hap.B^* were brought to Vietnam or Italy and spontaneously crossed with native varieties there, resulting in the gene flow of *OsEPFL1^Hap.B^*, but it is reasonable to speculate that *OsEPFL1^Hap.B^* was introduced from the outside of Japan, maintained in some indigenous varieties, and selected in the heterogeneous Kamenoo population.

“Bozu (NAR083)” is a landrace of Hokkaido, the northernmost part of Japan, and showed a deletion of 2,630 bp, including its half part downstream from the first intron (*OsEPFL1^Hap.E^* in **Supplementary Figure S2A**). According to a document (Nishio and Fujimaki, 2020), Bozu was originally selected as an awnless mutant from awned “Akage” in 1895. The genome structures of all six accessions (JRC017, NAR009, NAR010, NAR012, NAR013, and NAR014) registered as Akage and Bozu (NAR083, only one line in the NARO Genebank) were compared by phylogenetic analysis (**Supplementary Figures S4A, C**). Six Akage accessions were awned by functional *OsEPFL1^Hap.C^* (**Supplementary Figure S1**), with “JRC017 and NAR010” and “NAR009 and NAR012” forming two pairs each, which also showed some degree of genetic relationship (**Supplementary Figures S4A, C**). Of the remaining two accessions, NAR013 showed a low but still some degree of genetic relationship with these four accessions, while NAR014 showed no similarity to other Akage. These results indicate that Akage should be considered a heterogeneous group with a high degree of diversity, as is the case with Kamenoo. Next, we examined the relationship between Bozu carrying *OsEPFL1^Hap.E^* and Akage carrying *OsEPFL1^Hap.C^* and found a certain degree of relationship with the Akage pair (NAR009 and NAR012). *OsEPFL1^Hap.E^* is also shared by another Hokkaido landrace, “Sakigake”, which also showed some phylogenetic relationships with the Bozu and Akage pairs (NAR009 and NAR012) but did not form identical clades (**Supplementary Figure S4C**). These results lead us to speculate that *OsEPFL1^Hap.E^* has been shared and maintained in Hokkaido landraces with somewhat different genetic backgrounds. As an analogy of *OsEPFL1^Hap.B^*, we speculated the possibility that *OsEPFL1^Hap.E^* was introduced from outside of Japan and searched for lines with *OsEPFL1^Hap.E^* in RiceVarMap v2.0. There was no accession in this panel, and thus, *OsEPFL1^Hap.E^* is considered to be a mutation that occurred in Japan. Curiously, *OsEPFL1^Hap.E^* was found only in “Ishikari”, a modern cultivar, but not in “Bozu1”, “Bozu5”, and “Bozu6”, which are believed to be pure lines selected from Bozu, whereas these varieties showed no genome similarity with Bozu (**Supplementary Figures S4A, C**). In addition, almost all Hokkaido varieties bred after 1960 have *OsEPFL1^Hap.D^*, with a few exceptions (**Figure 2**). *OsEPFL1^Hap.D^* is the dominant haplotype in *indica* and tropical *japonica* varieties (**Supplementary Figure S2B**), but it is not found in almost all modern varieties except for Hokkaido (**Figure 2**), and thus *OsEPFL1^Hap.D^* should have been introduced from outside of Japan. It is interesting why the *OsEPFL1^Hap.D^* selection had been done during rice breeding in Hokkaido instead of *OsEPFL1^Hap.E^*. In this connection, the pure line selections from Bozu, Bozu1, Bozu5, and Bozu6 do not have *OsEPFL1^Hap.E^* but have *OsEPFL1^Hap.A^*, *OsEPFL1^Hap.D^*, and *OsEPFL1^Hap.B^*, respectively (**Supplementary Figure S1**). These observations suggest that the genomic region surrounding *OsEPFL1^Hap.E^* may contain some genes unfavorable for rice cultivation in Hokkaido.

### 3.3 The OsEPFL1 ortholog is not shared by some important crops such as wheat, barley, maize, and sorghum, even though it is shared by almost all grass plants

Next, we performed an *OsEPFL1* phylogenetic tree analysis in Poaceae and again examined the evolution of this gene and its relationship to cultivation/domestication. Previously, Takata et al. (2013) found a total of 12 genes as *EPF/EPFL* paralogs in rice and reported *OsEPFL2* as the gene with the highest structural similarity to *OsEPFL1*. More recently, Xiong et al. (2022) reported that *OsEPFL2* is also involved in rice awn formation. Therefore, we also collected *OsEPFL2* orthologs within Poaceae to distinguish each other. For this phylogenetic tree analysis, we used *Amborella trichopoda* as a representative of sister lineages of angiosperms, *Arabidopsis thaliana* as a representative of dicot plants, and banana (*Musa acuminata*) and pineapple (*Ananas comosus*) as monocot representatives (**Supplementary Table S4**). The top hit genes of *OsEPFL1* and *OsEPFL2* in these plants outside Poaceae were the same (**Supplementary Figure S5**), suggesting that *OsEPFL1* and *OsEPFL2* were differentiated after the establishment of Poaceae. Subsequently, we searched for corresponding genes in *Joinvillea ascendens*, which diverged from the common ancestor of Poaceae before its whole genome duplication, and *Pharus latifolius*, a sister lineage of the core Poaceae used to elucidate genome structure and gene duplication/loss dynamics in Poaceae (**Supplementary Table S4**). Phylogenetic analysis including all *EPF/EPFL* homologs showed that the two homologs in *Pharus latifolius* showed a clear correspondence with *OsEPFL1* and *2*, and one of *Joinvillea ascendens* corresponded to *OsEPFL1*, but the other one did not (**Supplementary Figure S5**). We used these homologs in *Pharus latifolius* and *Joinvillea ascendens* to distinguish *OsEPFL1* from *OsEPFL2* in further analysis.

**Figure 3:**
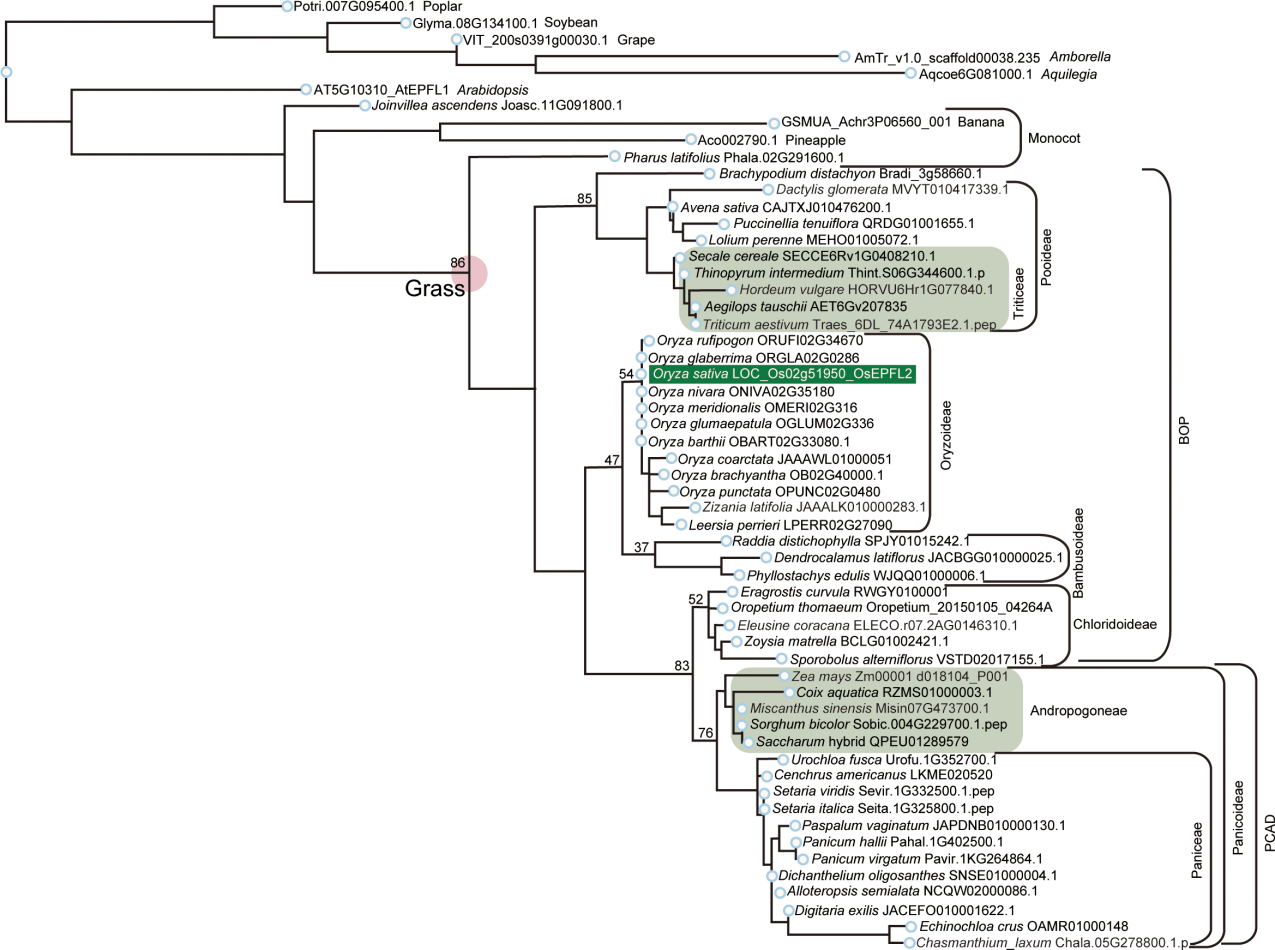
Phylogenetic analysis of *EPFL2* homologs in Poaceae. Phylogenetic tree of *EPFL2* homologs from 47 Poaceae plants, with non-Poaceae plants, *Amborella*, *Arabidopsis*, poplar, soybean, grape, banana, pineapple, and *Joinvillea*, are included as outgroups. *OsEPFL2* is surrounded by a dark green square. Light green squares indicate the *EPFL2* homologs of the plants that do not have an *EPFL1* homolog (except for Misin07G473700 in Andropogoneae). The number shown on the left shoulder of each clade is the bootstrap value.

**Figure 4:**
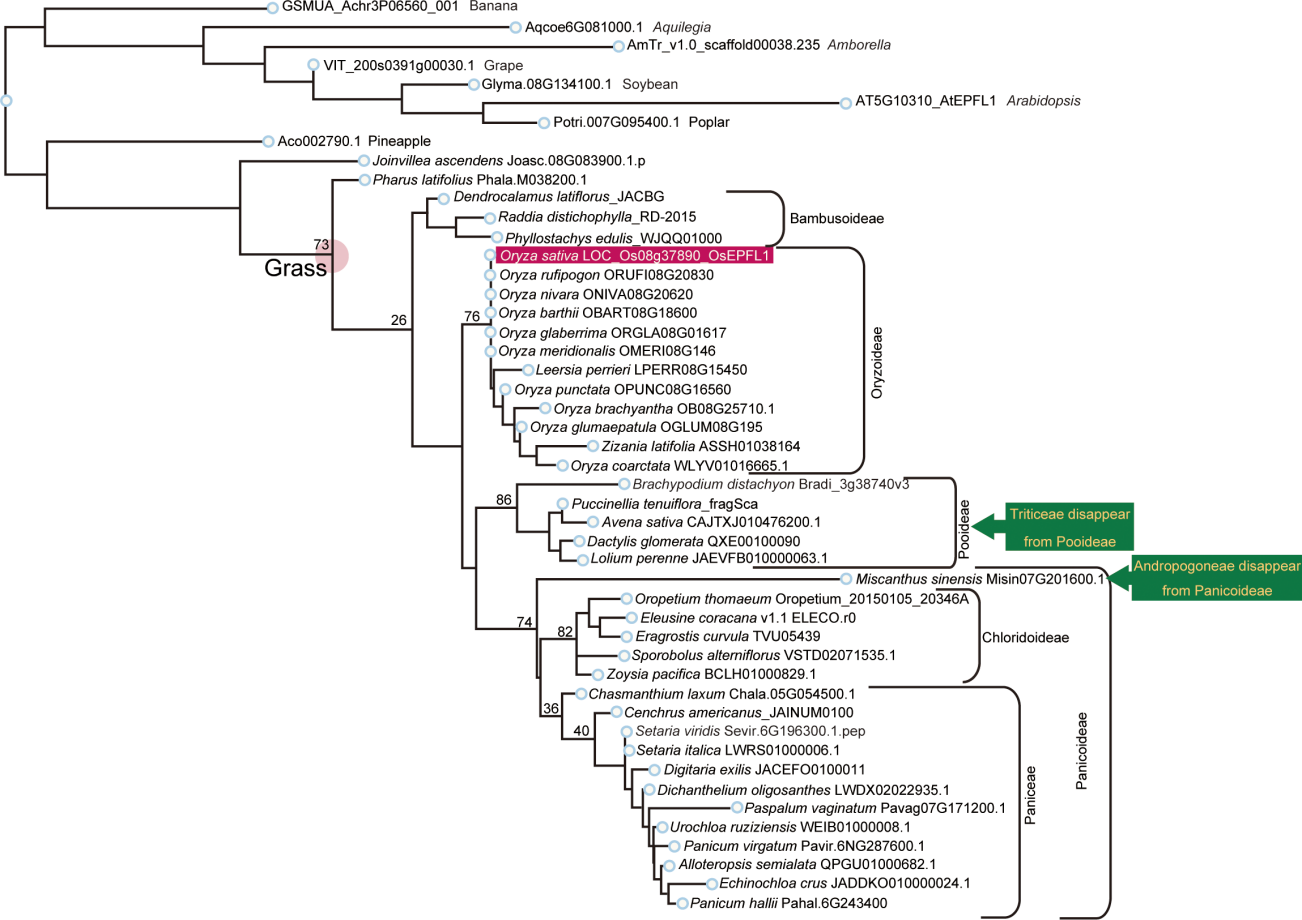
Phylogenetic analysis of *EPFL1* homologs in Poaceae. *EPFL1* homologs were analyzed as the same in **Figure 3**. *OsEPFL1* is surrounded by a red square. Triticeae and Andropogoneae, shown in yellow on a dark green background, represent clades that were present in the EPFL2 phylogenetic tree but missing in EPFL1 (except for Misin07G201600 in Andropogoneae).

**Figure 5:**
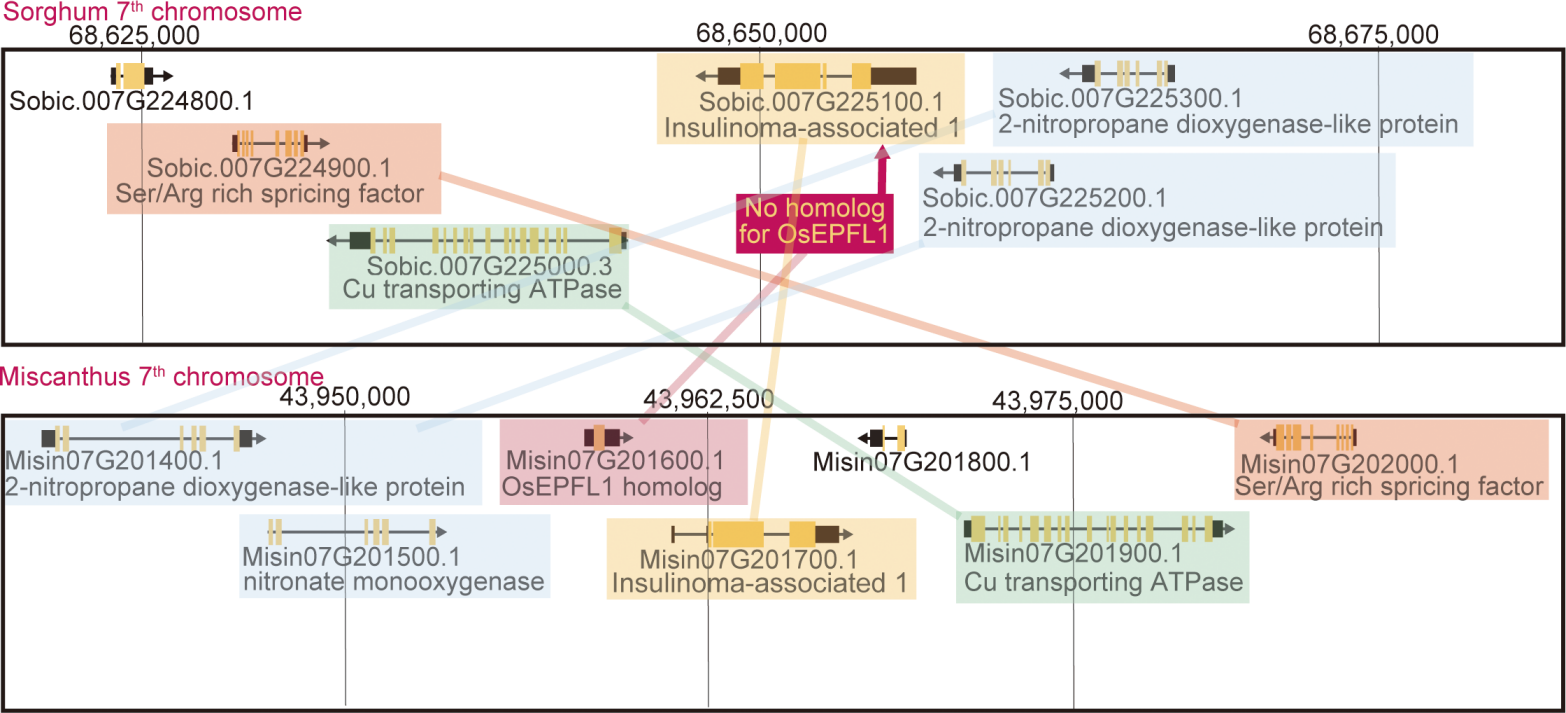
Synteny between the *Miscanthus EPFL1* region and its corresponding region in sorghum. The lines highlight the genes with syntenic relationships. The sorghum genome seems to lack only the *Miscanthus EPFL1* orthologous region.

We searched for orthologs of *OsEPFL1* and *OsEPFL2* in 47 species of Poaceae and found that *OsEPFL2* was found in all of them (**Supplementary Table S5**; **Figure 3**), whereas *OsEPFL1* was not found in five in Triticeae (wheat, barley, rye, goatgrass, and *Thinopyrum intermedium*) and four in Andropogoneae plants (maize, sorghum, sugarcane, and *Coix aquatica*) (**Supplementary Table S6**; **Figure 4**). Even when homologs are not found by TBLASTN for whole genome information, it is dangerous to easily conclude from this result that the gene is missing, but since all of these nine species are localized to only two specific tribes, it is very possible that the *OsEPFL1* ortholog loss has occurred during or after the establishment of the specific tribes. Subsequently, we compared the genome structure around *OsEPFL1* in *Miscanthus sinensis*, the only species in Andropogoneae with an *OsEPFL1* ortholog, with sorghum, which is closely related to *Miscanthus* but not has found the *OsEPFL1* ortholog. The *Miscanthus OsEPFL1* is located on the short arm of chromosome (Chr) 7, and the region showed good synteny with the long arm of Chr 7 of sorghum, but the *OsEPFL1* ortholog (Misin07G201600) was found to be missing from the sorghum genome (**Figure 5**). Furthermore, we performed a similar analysis between barley (without ortholog) and *Brachypodium* (with ortholog), but no syntenic region was found between these species (**Supplementary Figure S6**). This observation led us to speculate that the *EPFL1* region might be rearranged in the barley genome. Thus, we examined whether this genomic arrangement occurs frequently within the Poaceae between different subfamilies, rice (subfamily Oryzoideae) and *Brachypodium* (subfamily Pooideae). We found a high degree of homology between rice and *Brachypodium* genomes, despite the fact that the distance between these plants is far greater than that between *Brachypodium* and barley in the same subfamily, Pooideae (**Supplementary Figure S7**). Furthermore, the conservation of this genomic region is also observed between rice and *Pharus latifolius*, the common ancestor of the Poaceae (**Supplementary Figure S8**), showing that this region is not particularly unstable in the Poaceae and that the rearrangement of this region in Pooideae or Triticeae could be an individual situation of this lineage group.

### 3.4 Isolation of a novel awn-forming gene of moderately awned variety Omachi

There are some varieties that can develop long awn despite carrying the nonfunctional *OsEPFL1*, such as “Tamanishiki” with *OsEPFL1^Hap.A^* (**Figure 1P**) and “Hirayama” with *OsEPFL1^Hap.D^* (**Figure 1Q**). We speculated that the long awn is caused by an awn-forming gene(s) other than *OsEPFL1* and investigated other known awn-forming genes, such as *An-1* and *An-2* (Luo et al., 2013; Gu et al., 2015). Although most Japanese rice varieties have a mutator-like transposon on the promoter of *An-1*, which lacks gene function, some of these long-awned varieties have no such transposon (**Supplementary Figure 9**), indicating that *An-1* is also involved in awn formation in Japanese rice. With the addition of the *An-1* haplotype information, all 66 varieties judged to have vigorously awn development (**Supplementary Table S2**; **Figure 2B**) have *OsEPFL1^Hap.C^* and/or *An-1*, demonstrating that only these two genes can explain the vigorously awn formation of Japanese rice. However, there are still some awned varieties despite the functional deletion of *OsEPFL1* and *An-1*, such as "Omachi" (**Figures 1C, D**) and “Gohyakumangoku” (**Figures 1H, I**), which were evaluated as moderate (**Supplementary Table S2**). These varieties showed a large variation in the awn formation frequency and length (compare **Figures 1C, D, H, I**, respectively), indicating that the awn formation in these plants is regulated by a mechanism different from that of *OsEPFL1* or *An-1*. To study this novel mechanism of awn formation, we searched for loci involved in this phenomenon.

For this study, we obtained historical data on awn formation over a 12-year period from 1992 to 2003 at the Hyogo Prefectural Agricultural Experiment Station (Hyogo Panel). In these data, Nipponbare (KOB134) was consistently judged to have awn over a 12-year period, while Yamadanishiki (KOB118) was never judged to have an awn (**Supplementary Tables S7**, **S8**), although Nipponbare was judged as hardly in our panel (**Figures 1A, B**; **Supplementary Table S2**). There could be two reasons for such a difference between these observations. One is the difference in the growing environment (e.g., Kato, 1911; Takahashi et al., 1986b). The other is that Hyogo’s evaluation depended mainly on the awn formation of the spikelet at the top of the panicles, whereas our results were based on the whole part of the panicles. Even in one plant, there is a big difference in awn formation between the top (vigorously) and bottom (moderately) of the panicle, and this change gradually occurs from top to bottom. Varieties carrying the functional *OsEPFL1* and/or *An-1* can usually induce some awn length, even at the base of the panicle (**Figure 1**). In contrast, Omachi and other varieties with similar awn developmental characteristics can produce awns of some length at the top but hardly at the base of the panicle (**Figures 1B–D, F–I, L–N**). On the other hand, Yamadanishiki and other varieties with similar awn developmental characteristics do not develop awns even at the top of the panicle, or, even if they do, they develop only very short awns (**Figures 1E, O**). In this context, we considered that the Hyogo panel provides good material for the analysis of loci involved in the moderate awn formation of Omachi.

**Figure 6:**
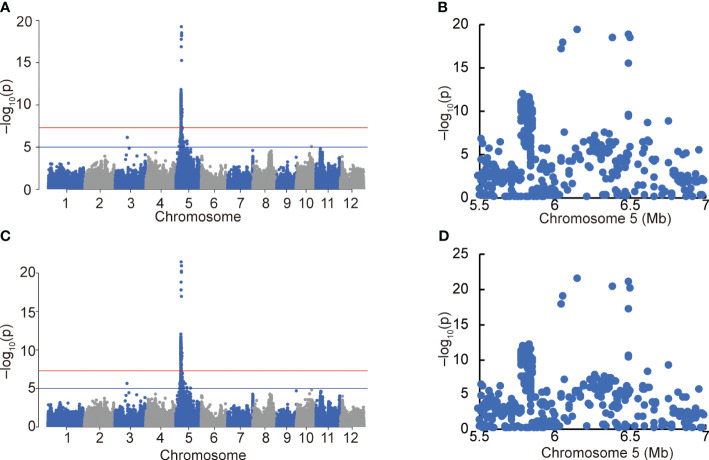
GWAS of awn formation and length using the Hyogo Panel. Manhattan plots of degree of awn formation **(A)** and awn length **(C)**. Red and blue horizontal lines indicate genome-wide significance (*p* = 5.0 × 10^−8^) and suggestive (*p* = 1.0 × 10^−5^) thresholds. Local Manhattan plots of the top peaks of **(A, C)** (**B**, **D**, respectively).

After excluding varieties carrying *OsEPFL1^Hap.C^* and *An-1* to eliminate the effect of these strong genes on awn formation, we used 153 varieties from the Hyogo Panel that overlap with our panel (**Supplementary Tables S7**, **S8**) and performed a GWAS using the degree of awn formation and “awn length” data (**Figure 6**). Because some lines were measured for only 1 year out of 12 years, the same analysis was performed on 128 varieties with data from multiple years of measurements (**Supplementary Figure S10**). The results were essentially the same in both cases, with only one strong peak in the short arm region of Chr. 5 (**Figure 6**; **Supplementary Figure S10**). In parallel, we also conducted a QTL analysis using back-cross inbred lines (BILs) between moderately awned “Reiho” (**Figure 1F**) and awnless “Yamadanishiki” (**Figure 1E**) and found a strong QTL in the same position (**Supplementary Figure S11**). In this candidate region, the awned Omachi and Reiho has the same haplotype of “Nipponbare”, whereas the awnless Yamadanishiki has a different haplotype.

**Figure 7:**
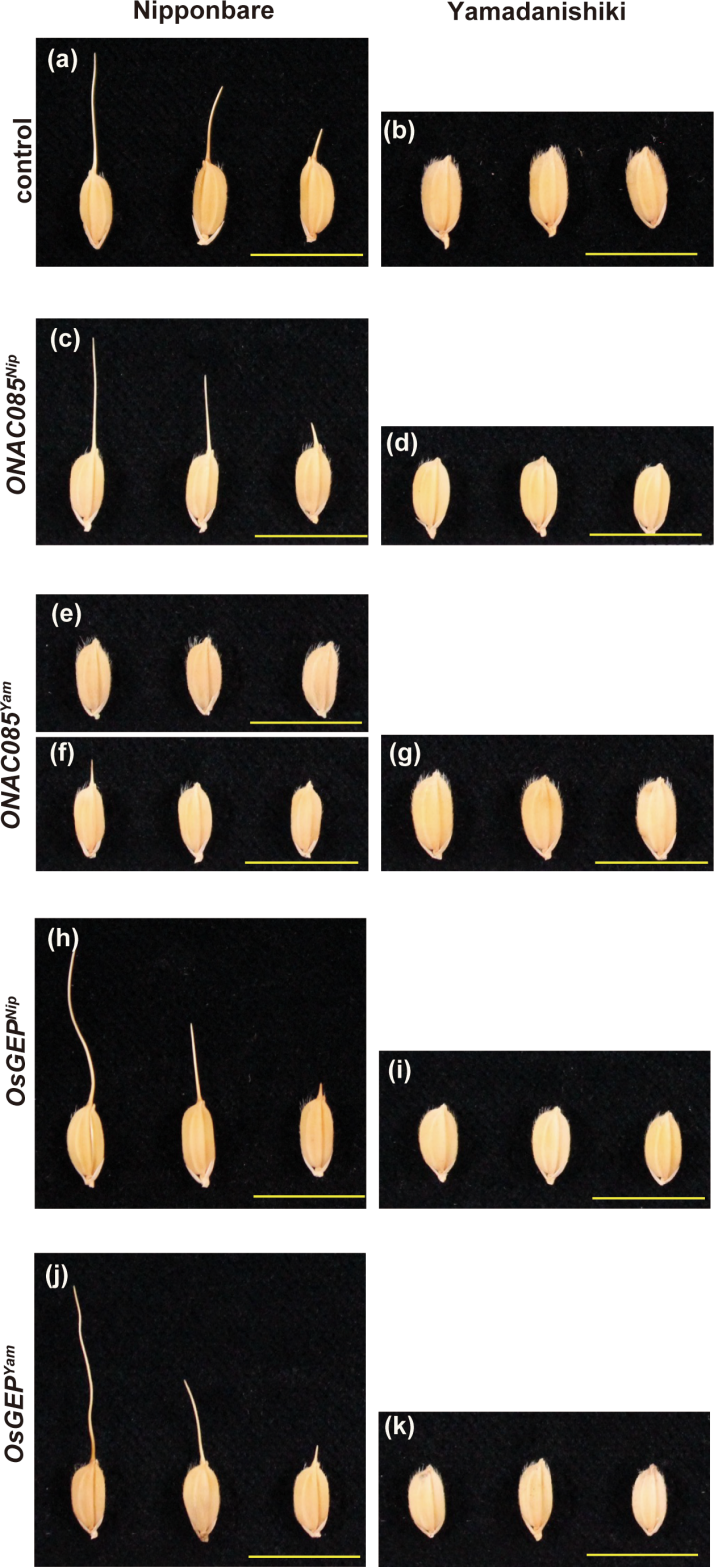
The effect of the two candidate genes on the awn development. Awns of the nontransformed Nipponbare and Yamadanishiki **(A, B)** and the transformants introduced with *ONAC085^Nip^* **(C**, **D)**, *ONAC085^Yam^* **(E–G)**, *OsGEP^Nip^* **(H**, **I)**, and *OsGEP^Yam^* **(J**, **K)**. Introduction of *ONAC085^Yam^* into Nipponbare attenuated the awn formation completely **(E)** to severely **(F)**.

To identify the causative gene among the DNA polymorphisms within the peak region of Chr. 5, we first narrowed down the 22 polymorphisms (nine genes) that were predicted to alter the protein structure using snpEff and that were −log_10_(*p*)>5 in the GWAS results (**Supplementary Table S9**). Among nine genes, five genes that RAP or MSU predicted not to be genes were excluded due to low reliability, narrowing the list to the four genes (four polymorphisms) shown in **Supplementary Table S10**. Based on comprehensive studies on these four genes, such as the expected impact of the substituted amino acids on gene function (**Supplementary Figure S12**), tissue specificity of expression, and responsiveness of expression against internal/external stimulation (**Supplementary Figure S13**), we selected two candidates *ONAC085*/Os05g0194500/LOC_Os05g10620 and *O*-sialoglycoprotein endopeptidase (*OsGEP*)/Os05g0194600/LOC_Os05g10630. We introduced the entire genomic regions of these genes into Nipponbare (awned) and Yamadanishiki (awnless) by transformation (**Figure 7**). The awn formation of Nipponbare transformed with Yamadanishiki *ONAC085* (*ONAC085^Yam^*) was strongly attenuated (**Figures 7E, F**; **Supplementary Figure S14**), while all other plants transformed with *ONAC085^Nip^* or *OsGEP^Yam/Nip^* showed no change in awn formation.

**Figure 8:**
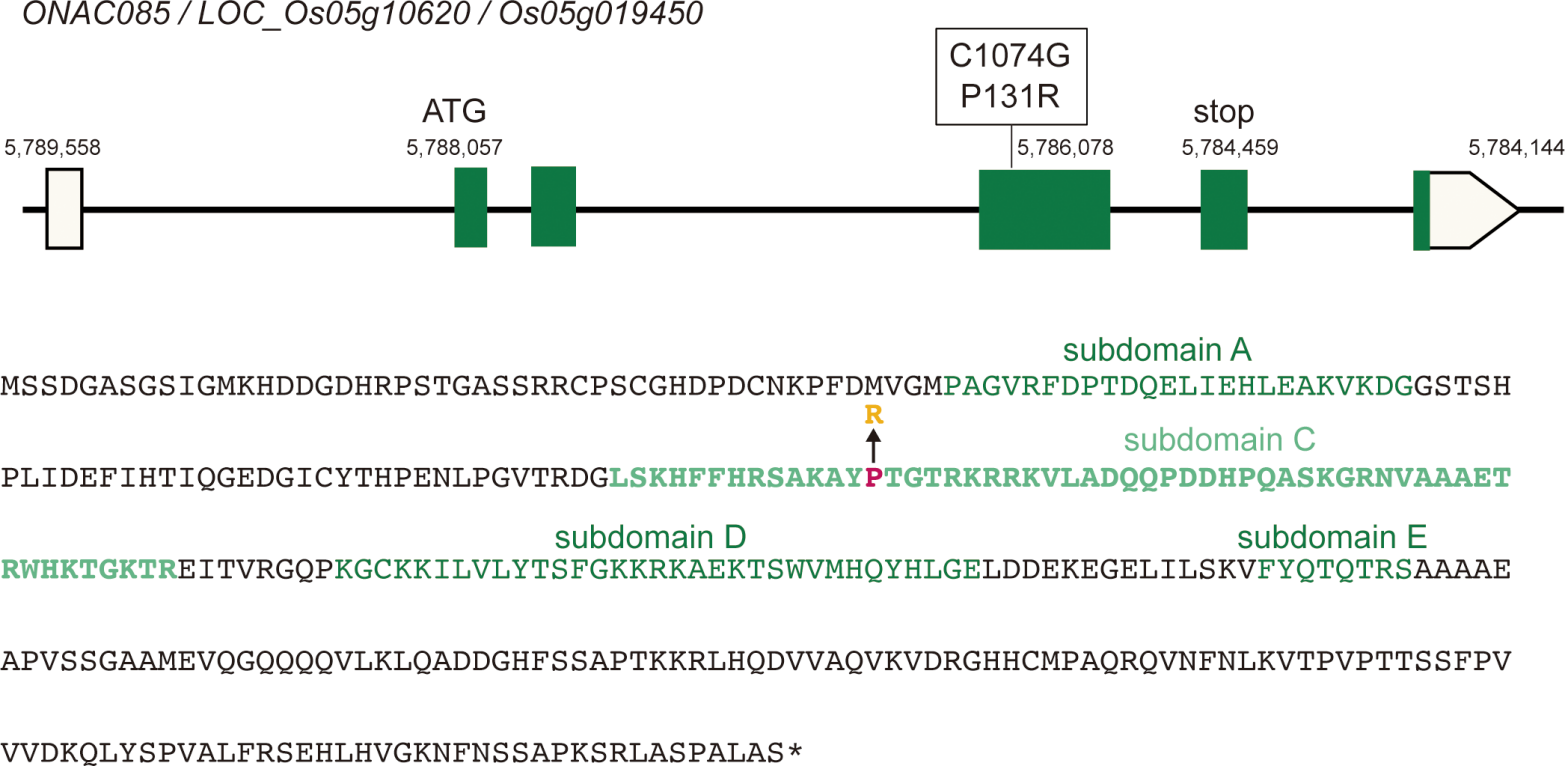
*ONAC085* structure and its polymorphisms. Top: The reference (Nipponbare) structure of *ONAC085* and DNA polymorphism found in Japanese rice varieties such as Yamadanishiki. Bottom: The ONAC085 amino acid sequence. Mutation of C1074G caused P131R amino acid substitution. There are five highly conserved subdomains in NAC-type transcription factors **(A–E)**, where P131 is present in subdomain C, which is predicted to be a DNA-binding site.

In comparison to the Nipponbare reference gene, the *ONAC085^Yam^* product has arginine for the 131st proline in subdomain C, which is located in the DNA-binding site of the NAC transcription factor (**Figure 8**). Subsequently, we conducted the phylogenetic analysis of this amino acid substitution and found that almost all homologs in both monocot and dicot have threonine, whereas only *Oryza* species with the AA genome, including *O. sativa* has a proline, while other Oryzeae species such as *Zizania palustris*, *Leersia perrieri*, and *O. brachyantha* with FF genome have threonine, as like other grass members (P1 in **Supplementary Figure S15**), suggesting that the substitution from threonine to proline has occurred during the evolution of *Oryza* species. The same substitution is found in sorghum and sugarcane, which belong to Andropogoneae, and should occur independently from *Oryza* species (P2 in **Supplementary Figure S15**), suggesting that this amino acid substitution may have conferred a novel function to this protein. In these backgrounds, the Yamadanishiki haplotype has a further change of this proline residue to another amino acid, arginine, which may have further altered the function of the new type of *ONAC085* that appeared in the AA genome. In parallel with these analyses, we also examined the segregation of awn formation by using the reciprocal crossed F2 population and found 37.5% (59/157) of the “Yamadanishiki × Reiho” F2 plants were awned, while 30.3% (53/175) of the “Reiho × Yamadanishiki” F2 plants were awned, indicating that the awnless phenotype is inherited as a dominant manner, corresponding to the observation that the haplotype of Yamadanishiki dominantly inhibited the awn formation in Nipponbare.

## 4 Discussion

### 4.1 Awn was an important organ for cultivation in the high latitudes of Japan

According to the first nationwide survey of rice varieties in Japan, about one-fourth of the total planted area in Japan at the beginning of the twentieth century was occupied by awned varieties (**Table 1**). Before the twentieth century, there were no national statistics on rice varieties in Japan, but some descriptions show that more than half of the varieties had an awn (e.g., **Supplementary Table S1**). In addition, there are also many descriptions of the selection of various awnless varieties in Japan around the beginning of the twentieth century (Nishio and Fujimaki, 2020), and the planted area of newly selected awnless varieties in this period accounted for more than 60% of the total planted area (**Table 1**). From these facts, we can assume that nearly half or more of the rice varieties grown in Japan before the twentieth century had an awn.

The regional distribution of *OsEPFL1^Hap.C^* was particularly biased toward high-latitude regions (**Figure 2A**). With regard to this tendency, Kato (1911) speculated that the awns increase the evaporation of water, as a result of which grain development is facilitated in areas of low temperature and high humidity. Sato and Takahashi (1984) compared transpiration, respiration, and photosynthesis between intact panicles and those with the awn artificially removed and reported that these values were reduced in the awnless panicle. Their experiment measured the difference between intact panicles and panicles with the awn removed, which can cause some secondary effects. In this study, we directly measured the activity of the awn itself and found only transpiration but not respiration (**Table 2**), which indicates that awns contribute greatly to transpiration during grain development. The awn of barley and wheat has stomata, which acts as a photosynthetic organand contributes to grain filling during development (Guo and Schnurbusch, 2016; Rebetzke et al., 2016; Huang et al., 2021). Some reports have interpreted rice awn as having stomata, but detailed observations of rice awn morphology have shown the absence of stomata in rice awn (Tatsumi and Kono, 1972). Therefore, when discussing the physiological role of rice awn, it is necessary to make a clear distinction between barley, wheat awn, and rice awn. During the ripening process of rice grains, the moisture content of the grains gradually decreases toward maturity (Brinkhoff et al., 2023), while water is produced in the reaction to synthesize starch, which accounts for 80% of the total mass of rice grains, from sucrose. It is therefore necessary to release water from the grain efficiently. Transpiration occurs easily in a sunny, high-temperature environment, but in a low-temperature environment with rainfall, which is common in the cold summer in the Tohoku and Hokkaido regions, transpiration is inhibited due to extremely high relative humidity (e.g., Singh and Sasahara, 1981). The amount of transpiration from the grain is ~400 µmol s^−1^ per grain, and the amount from the awn reaches about 30% of the total grain transpiration due to its unique high surface area structure.

Given the physiological functions of the awn described above, it is reasonable to assume that rice varieties with a long awn were dominant in the high latitudes of Japan. In fact, there are many agricultural documents from the early nineteenth century describing the superiority of awned varieties over awnless varieties in cold-weather cultivation (e.g., Sato, 1829, Tamura, 1841, Izumi, 1894, Mine, 1898; Takahashi, 1915). In addition, when severe cold damage occurred several times in Japan’s high latitudes around 1900, the agricultural experiment stations in the Tohoku region issued guidelines suggesting that awned varieties should be grown against cold damage (reviewed by Yasuda, 1955). For example, all 11 varieties that yielded more than 350 kg/10 in the cold summer of 1905 were awned, while 11 of the 12 varieties that yielded less than 75 kg/10 were awnless (Iwate Prefectural Agricultural Experiment Station, 1907a). This report also mentioned that when the panicle emergence time was affected by a long period of rain, the yield of awnless varieties was significantly reduced. These results clearly support the above discussion that transpiration through the awn is important for grain development. Based on these results, the Iwate Prefectural Agricultural Experiment Station selected 11 awned species as superior species out of more than 100 species (Iwate Prefectural Agricultural Experiment Station, 1907b). There is an interesting fact that shows the importance of awn at that time. An awned variety, “Togo” with *OsEPFL1^Hap.C^*, was selected in 1901 from the awnless variety, Ooba with *OsEPFL1^Hap.A^*, which was once selected as an awnless variety, and “Moritawase”, which can develop a longer awn by *OsEPFL1^Hap.C^* and functional *An-1*, was reselected in 1913 from Ooba (**Supplementary Figure S1**, Nishio and Fujimaki, 2020). This clearly shows that “progress in cultivation and breeding is accompanied by a reduction in awning” is not correct, even though the selection of awned varieties from awnless ones is unlikely based on current knowledge of genetics.

In explaining why awn was actively removed in the early twentieth century despite its contribution to cold tolerance, Takahashi et al. (1986a) pointed to the sensitivity of awned varieties to nitrogen fertilizer, which was rapidly introduced in the early twentieth century. According to them, awned varieties had a high risk of lodging and poor disease and pest resistance under high nitrogen conditions. In addition, the introduction of modern breeding methods improved cold tolerance and, there was no longer a need to rely on awned varieties. An agricultural book published in 1841 stated: “It is outrageous for a farmer to despise the merits of the awning and to be reluctant to grow awned varieties because of their troublesome character”. This book shows that even then, farmers were caught between the risk of cold damage and the desire to grow awnless varieties.

### 4.2 Domestication of Poaceae plants and its relationship with EPFL1

**Figure 9:**
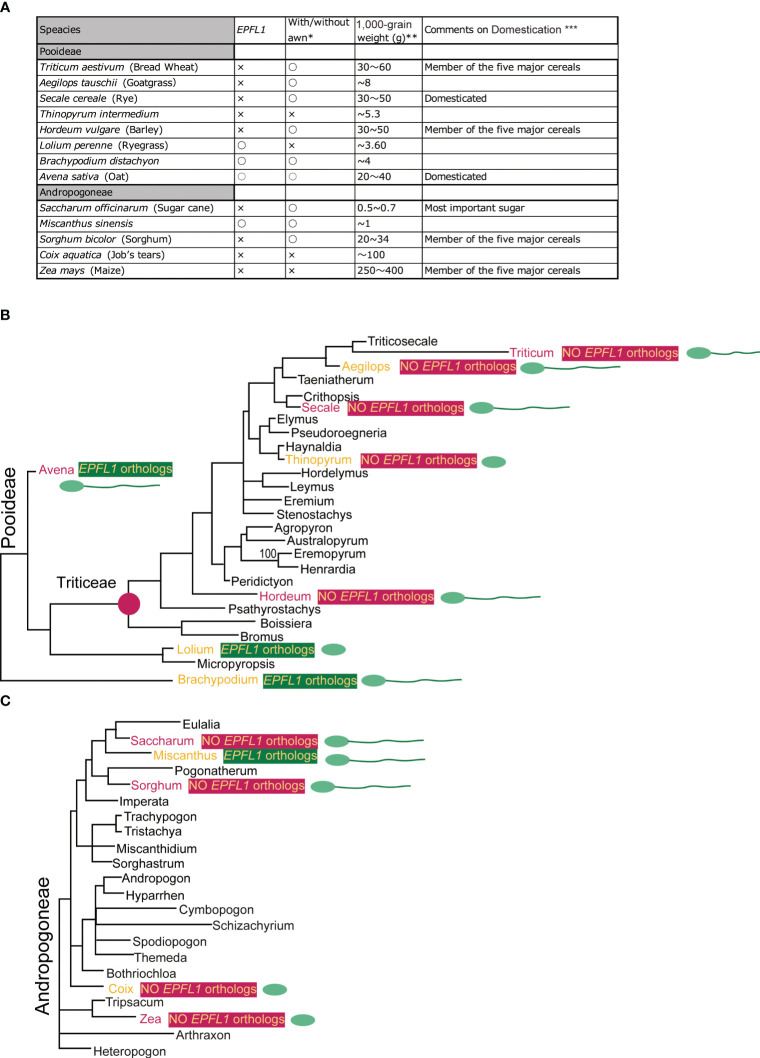
Comparison of with/without *EPFL1*, awn formation, and 1,000-grain weight in plants belonging to Pooideae and Andropogoneae **(A)** and their phylogenetic relationship **(B**, **C)**. In **(A)**, “with/without awn formation” and “1,000-grain weight” refers to the following locations: https://herbaria.plants.ox.ac.uk/bol/plants400/, http://agroatlas.ru/en/search/, https://seedidguide.idseed.org/, https://en.wikipedia.org/wiki/, https://gobotany.nativeplanttrust.org/search/, http://dev.floranorthamerica.org/, and https://www.agriexam.com/test-weight-of-different-crops. ^*^Domesticated plants often do not have an awn, but where there are wild and domesticated plants of the same species, the presence/absence of an awn in the wild-type plant is indicated, with only clear awn formation marked with “○” and no awn formation or uncertainty marked with “×”. ^**^Since 1,000-grain weight varies greatly with variety and growing conditions, the specific numbers here are not as meaningful but are used to indicate a trend. ^***^The five major crops (wheat, barley, sorghum, and maize; excluding rice), rye, oats, and sugarcane are annotated. The phylogenetic trees of Pooideae **(B)** and Andropogoneae **(C)** are derived from Bouchenak-Khellad et al. (2008). The seven plants commented on for domestication are marked in red, and those with/without *EPFL1* are marked in yellow on a dark green/dark pink background. The seven plants (wheat, rye, barley, oat, sugar cane, sorghum, and maize) that were commented on for domestication **(A)** are shown in red. Other plants (goat grass, Thinopyrum, ryegrass, Brachypodium, Miscanthus, and Coix) are indicated in orange. The presence or absence of an awn is indicated schematically in the spikelet.

In rice, disruption of *OsEPFL1* causes awn reduction, but no relationship between *EPFL1* and awn formation has been observed in other grass plants. In fact, in Tririceae and Andropogoneae, some plants lacking *EPFL1*, such as wheat, barley, and sorghum, can form long awns, whereas *Lolium perenne* and *Avena sativa*, which have functional *EPFL1*, do not form long awns (**Figure 9**), although their wild species can develop awns. These observations lead us to speculate that *EPFL1* was originally unrelated to awn formation in the Poaceae but may have become involved in awn formation in rice by chance. A similar phenomenon has recently been reported for *DAI* involved in awn formation in sorghum (Takanashi et al., 2022). According to this report, a new *DAI* gene created by gene duplication of *DAI*^ori^, which was originally unrelated to awn growth, is now involved in the awn formation process.

Furthermore, we found no *EPFL1* homolog in wild maize (*Zea luxurians*) or wild wheat (*Triticum dicoccoides*), suggesting that *EPFL1* is not involved in the domestication process of these crops. Nevertheless, *EPFL1* orthologs have been lost in all four of the five major cereal crops (maize, wheat, barley, sorghum) except rice, which belongs to two different genera, Tririceae and Andropogoneae, which reminds us of the link between *EPFL1* and domestication. There are also domesticated plant species in other genera (e.g., *Panicum*, *Setaria*, *Elusine*, *Avena*, etc.), all of which retain functional *EPFL1* orthologs (**Figure 4**). With regard to oats carrying the functional *EPFL1* ortholog, it has been discussed that the domestication of oats, which occurred in the same area as wheat and barley, did not progress as rapidly as that of wheat and barley because increases in grain size and yield were not achieved smoothly (Oxford University Plants 400, Preece et al., 2016). This leads us to speculate about a link between *EPFL1* and the increase in yields. We compared the 1,000-grain weights of plants with and without *EPFL1* orthologs (**Figure 9**) and found that plant species without *EPFL1* orthologs tended to have higher values, while *Miscanthus*, *Lolium*, and *Brachypodium* with *EPFL1* had lower values. Although this may also be related to the domestication stage, *Coix*, which is not well domesticated, was extremely high at 100 g of 1,000-grain weight (**Figure 9A**), supporting the above hypothesis. In the case of rice, Jin et al. (2016) reported that *OsEPFL1/GAD1* has an effect on grain formation, and its loss of function increases rice yield, which also supports the above possibility. *OsEPFL1/GAD1* is a pleiotropic regulator of both awn formation and grain number/length in rice, whereas there is no association between *EPFL1* and awn formation in grass plants other than rice. If only the involvement of *EPFL1* in grain number/length is conserved in other plants, this would explain the relationship between *EPFL1* loss and domestication in grasses. In any case, the function of *EPFL1* in Poaceae and domestication could be interesting topics to study from different perspectives in the future.

### 4.3 Isolation of the awn gene in response to changes in the panicle position and external environment

We have isolated the causal gene involved in unstable awn formation, which is sensitive to panicle position and environmental changes, as seen in Omachi (**Figure 7**). In most cases, mutations involved in awn formation are due to a loss of gene function, but in this case, the mutant gene product was predicted to have an inhibitory function on awn formation. Given that the mutation results in a highly conserved amino acid substitution in the DNA-binding region of the NAC transcription factor (**Figure 8**; **Supplementary Figure S15**), it is possible that the mutant protein may have some adverse effect on the interaction between normal *ONAC085* and the *cis*-DNA sequence of target genes. Some members of the NAC transcription factors are known to be involved in seed development by regulating cell division and stress-response gene expression. *ONAC020*, *ONAC023*, and *ONAC026* are specifically expressed during rice seed development and are involved in seed size determination by regulating both cell division and cell expansion (Mathew et al., 2016). On the other hand, NAC transcription is also involved in plant water stress tolerance (Yang et al., 2022). *ONAC085* is preferentially expressed in developing floral organs, and its expression is rapidly upregulated by water stress-related treatments such as drought, ABA, and jasmonic acid (**Supplementary Figure S13**). As mentioned above, the physiological significance of awn is to promote transpiration during grain development; the induction of awn formation by *ONAC085* in response to the external environment is a reasonable scenario. This gene can be used as a clue to elucidate the molecular mechanism of water stress tolerance during grain development.

## Data availability statement

The datasets presented in this study can be found in online repositories. The names of the repository/repositories and accession number(s) can be found in the article/**Supplementary Material**. The vcf data are available in zenodo at https://doi.org/10.5281/zenodo.10990900.

## Author contributions

MS: Data curation, Formal analysis, Funding acquisition, Investigation, Methodology, Software, Validation, Visualization, Writing – original draft, Writing – review & editing. HY: Data curation, Formal analysis, Funding acquisition, Investigation, Methodology, Validation, Writing – original draft, Writing – review & editing, Resources, Software. SY: Formal analysis, Investigation, Methodology, Resources, Writing – original draft. MK: Investigation, Resources, Writing – original draft. EK: Investigation, Resources, Writing – original draft. MM: Investigation, Resources, Writing – original draft, Conceptualization, Data curation, Formal analysis, Funding acquisition, Methodology, Project administration, Supervision, Validation, Visualization, Writing – review & editing. SK: Data curation, Formal analysis, Funding acquisition, Investigation, Methodology, Validation, Visualization, Writing – original draft, Writing – review & editing.
