# Supplementary material for: Redefining awn development in rice through the breeding history of Japanese awn reduction
Source: Front Plant Sci. 2024 May 16;15:1370956. doi: 10.3389/fpls.2024.1370956 (PMC11137238; doi:10.3389/fpls.2024.1370956)
Supplement: Supplementary file 1 [file DataSheet_1.pdf]

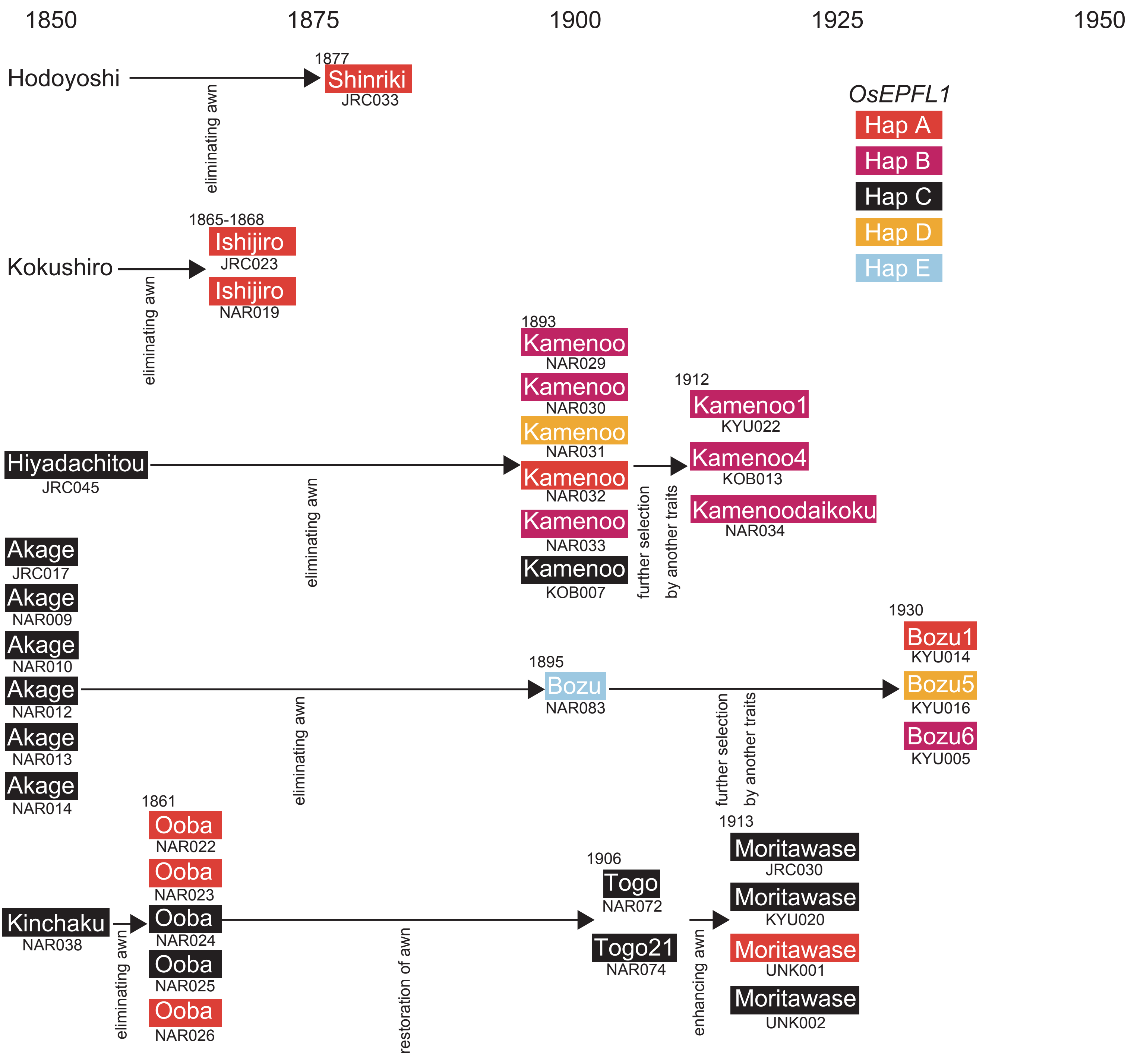


Sup. Fig. 1 Chronology of awnless selection in Japan during the 19th and 20th centuries

The breeding process of the awnless varieties in Table 1, "Shinriki, Ooba, Ishijiro, Kamenoo, and Bozu", which were selected from the awned varieties carrying functional *OsEPFL1*$^{Hap.\ C}$.

Supplemental Figure 1 Suganami et al.

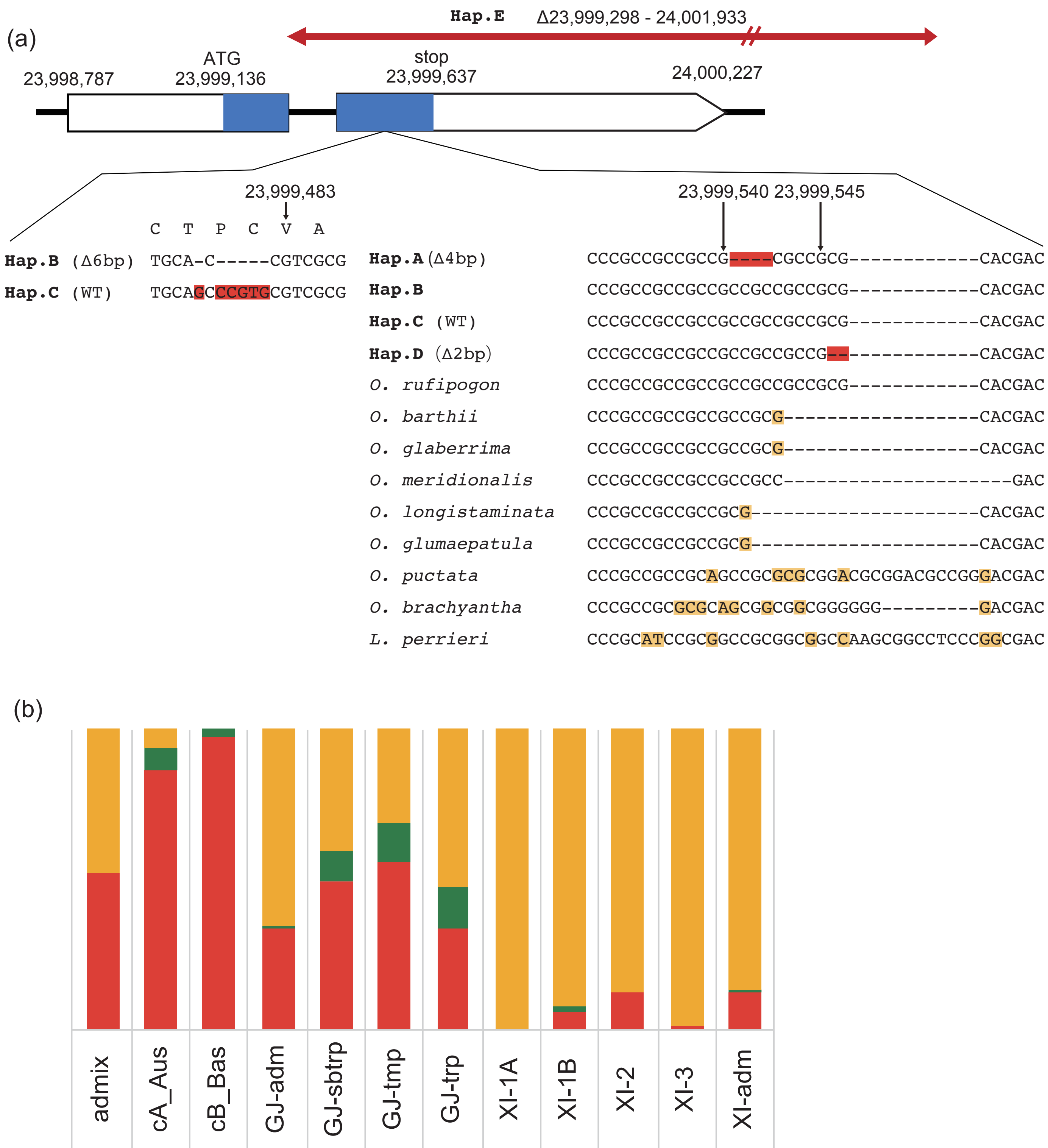


| | admix | cA_Aus | cB_Bas | GJ-adm | GJ-sbtrp | GJ-tmp | GJ-trp | XI-1A | XI-1B | XI-2 | XI-3 | XI-adm |
|---|---|---|---|---|---|---|---|---|---|---|---|---|
| D | 29 | 8 | 0 | 39 | 35 | 60 | 148 | 136 | 99 | 207 | 397 | 366 |
| C | 0 | 10 | 1 | 1 | 9 | 25 | 40 | 0 | 2 | 0 | 1 | 4 |
| A | 32 | 114 | 45 | 20 | 43 | 109 | 96 | 0 | 6 | 29 | 4 | 52 |

Sup. Fig. 2 *OsEPFL1* structure and distribution of its different haplotypes

(a) Gene structure of *OsEPFL1* with polymorphisms among *Oryza* spp. and *Leersia perrieri* (*L. perrieri*). (b) Haplotype frequencies of Hap.A, C, and D among subpopulations of the IRRI_3K panel (https://snp-seek.irri.org/).

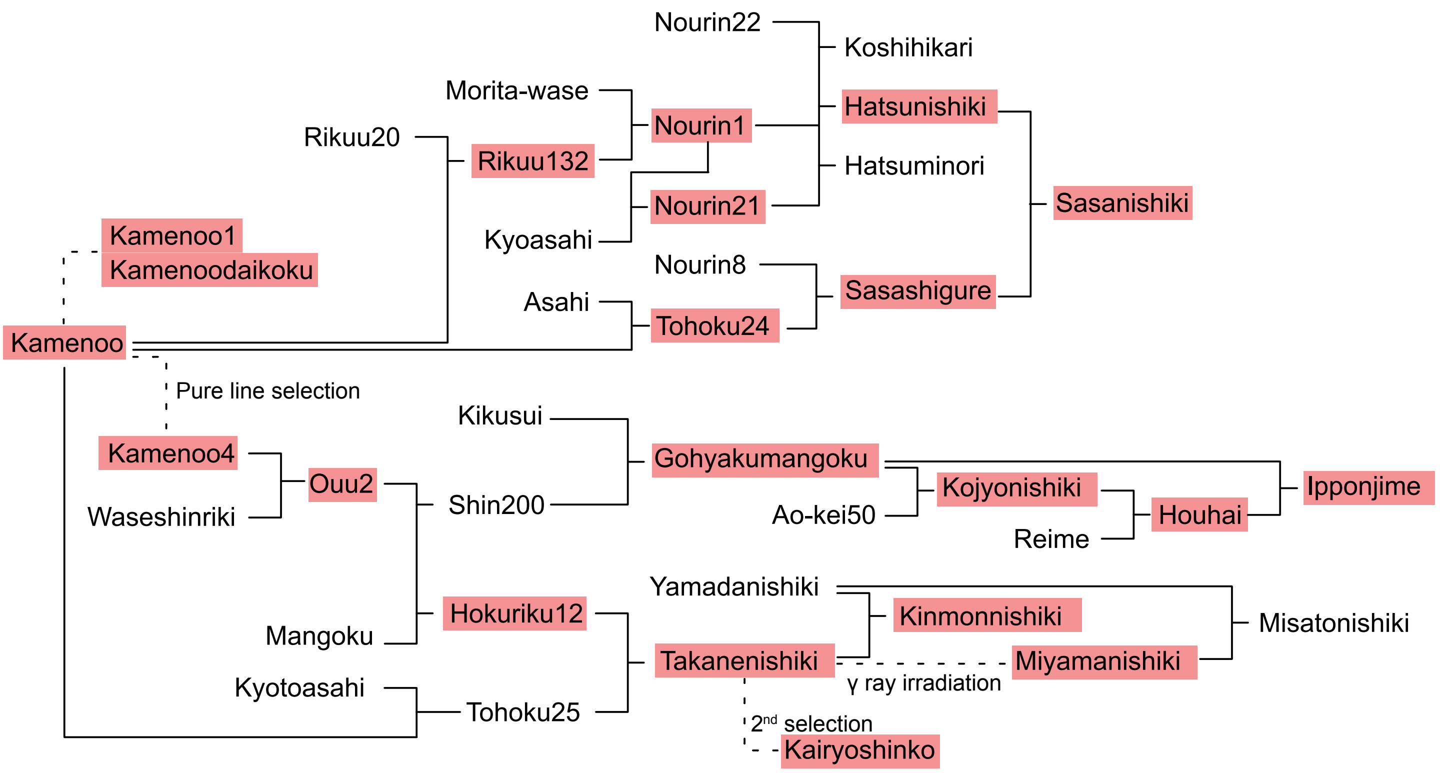


Sup. Fig. 3 Pedigree chart with “Kamenoo” as the origin

Varieties marked in red have *OsEPFL1*$^{HapB}$.

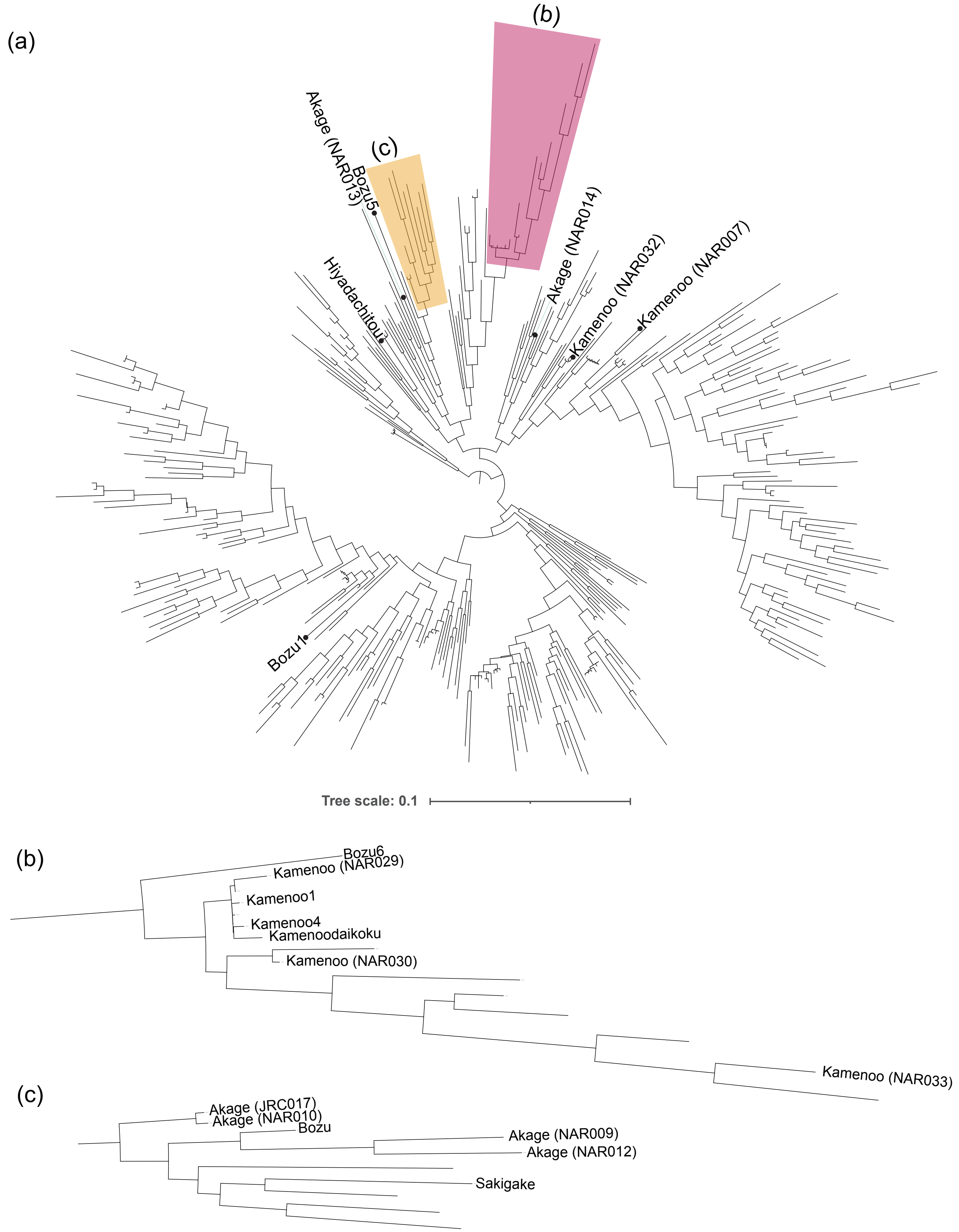


Sup. Fig. 4 Phylogenetic relationship of “Kamenoo” and “Bozu” and their relatives

Where the discussed varieties are close together, the areas are highlighted in red and yellow and enlarged in (b) and (c).

Supplemental Figure 4 Suganami et al.

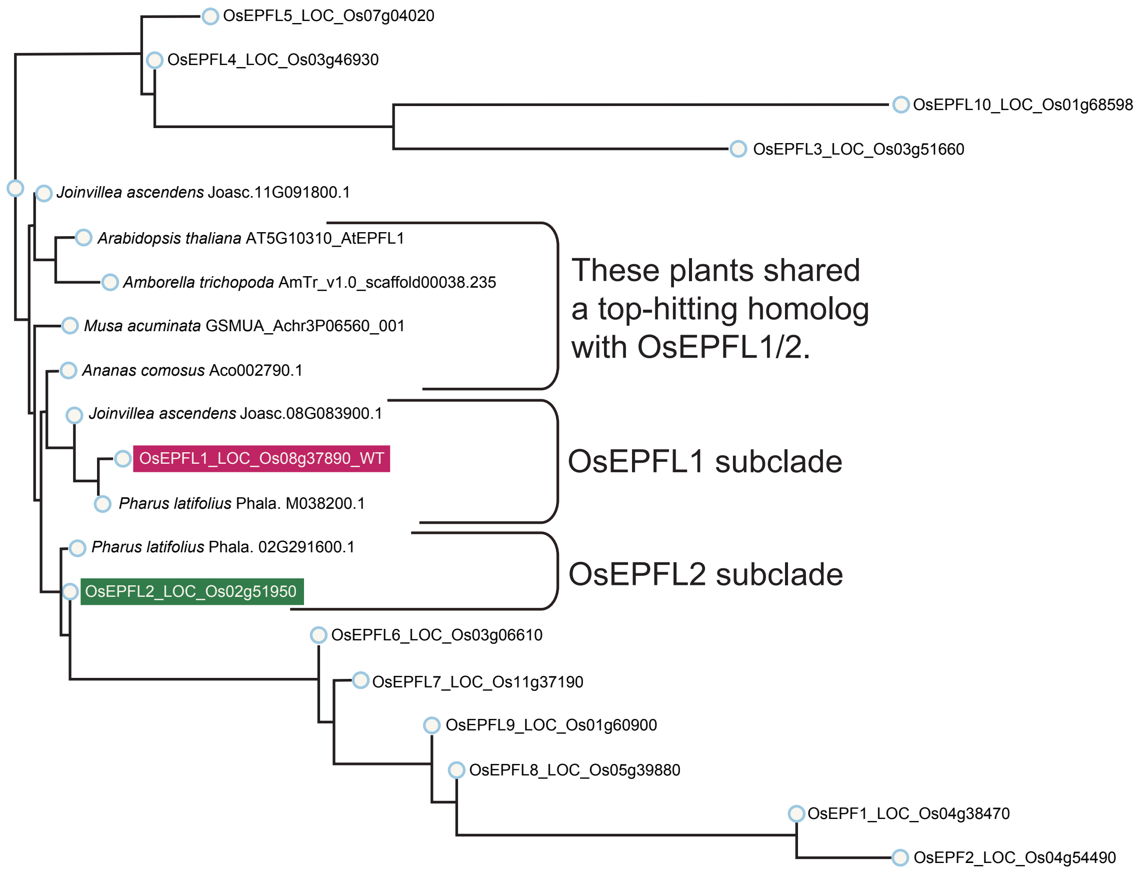


Sup. Fig. 5 Phylogenetic analysis of the EPF/EPFL proteins in rice

Phylogenetic tree of all 12 EPF/EPFL homologs in rice, together with the most closely related EPFL1/2 homologs in *Amborella trichopoda*, *Arabidopsis thaliana*, banana (*Musa acuminata*), and pineapple (*Ananas comosus*), as well as the EPFL1 and EPFL2 homologs in *Joinvillea ascendens* and *Pharus latifolius*.

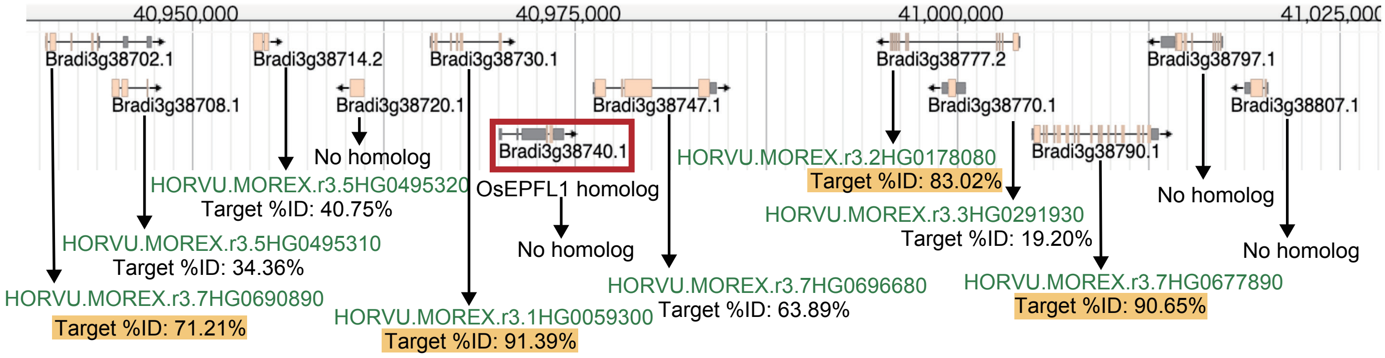


Sup. Fig. 6 Synteny between the *Brachypodium EPFL1* region and its corresponding region in barley

No genomic region in barley was found to be syntenic to the region surrounding *Brachypodium EPFL1* (Bradi3g38740). Therefore, for the genes adjacent to *Brachypodium EPFL1*, barley genes with high homology are presented individually. Four of the 12 genes (highlighted in yellow) had homologies greater than 70%.

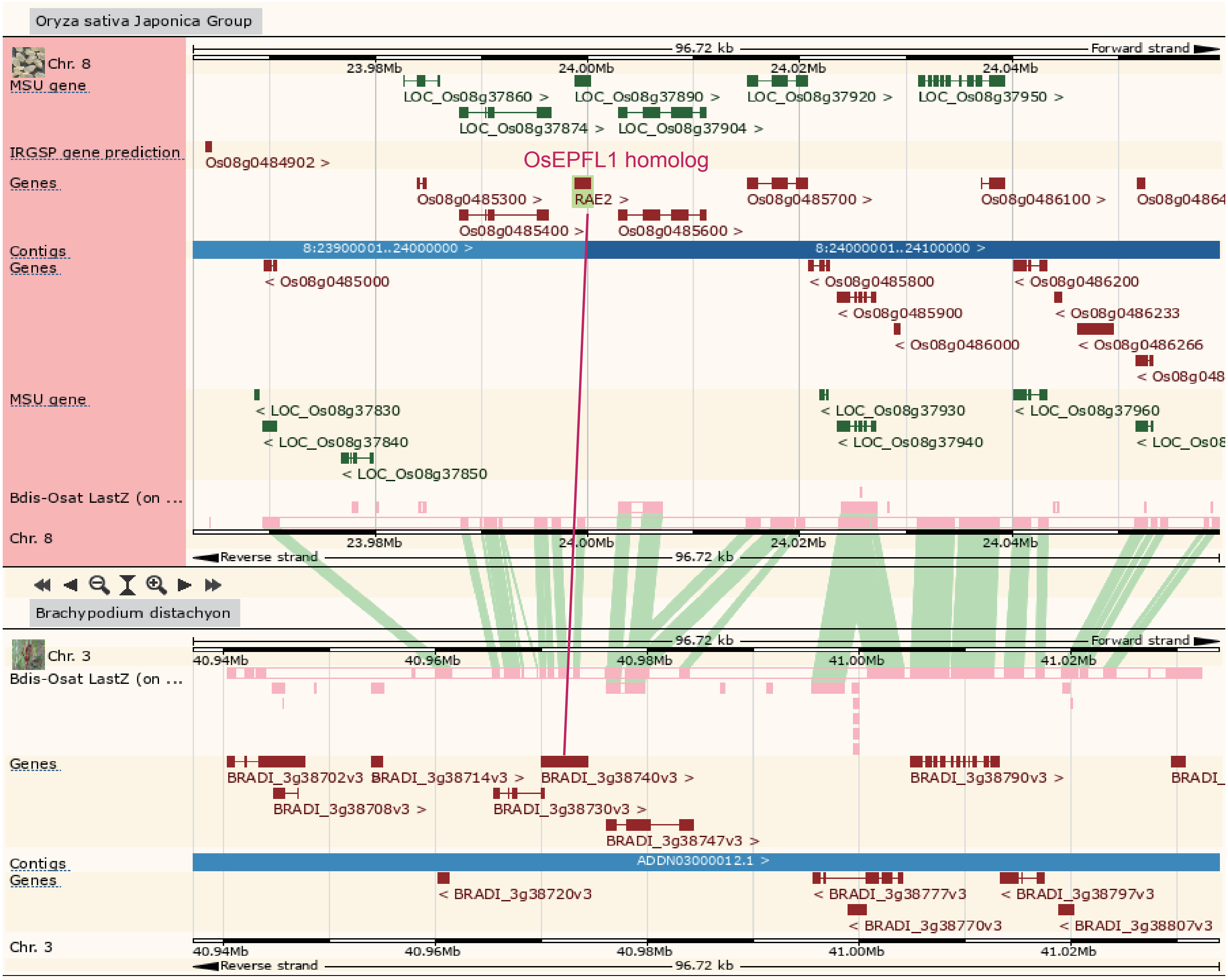


Sup. Fig. 7 Synteny between the rice *OsEPFL1* region and its corresponding region of *Brachypodium distachyon*

This synteny analysis was conducted in Ensembl Plants (https://plants.ensembl.org/index.html).

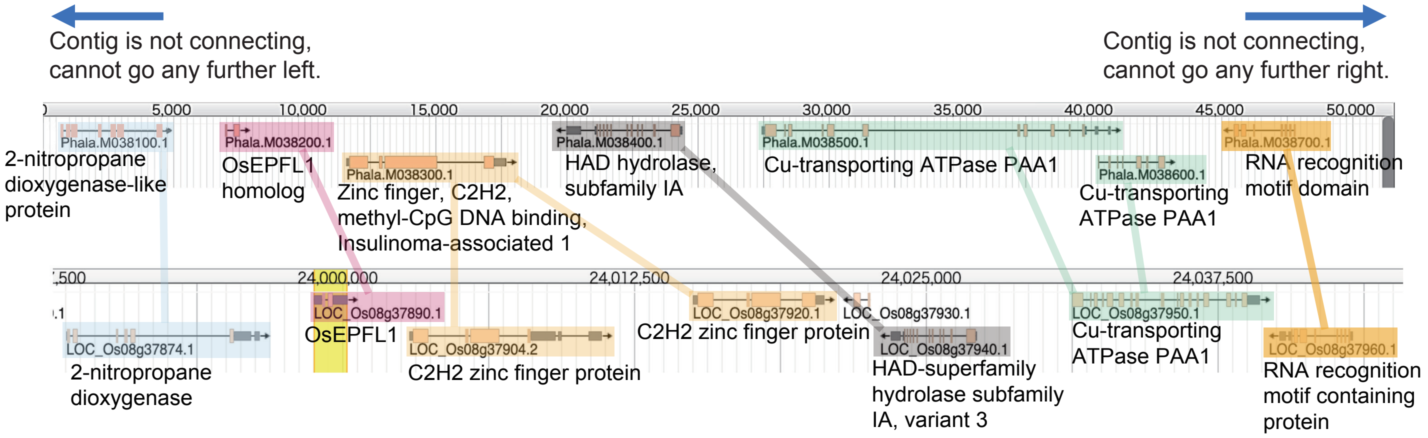


Sup. Fig. 8 Synteny between the rice *OsEPFL1* region and its corresponding region in *Pharus latifolius*

The genome browser images were obtained from Phytozome (https://phytozome-next.jgi.doe.gov/).

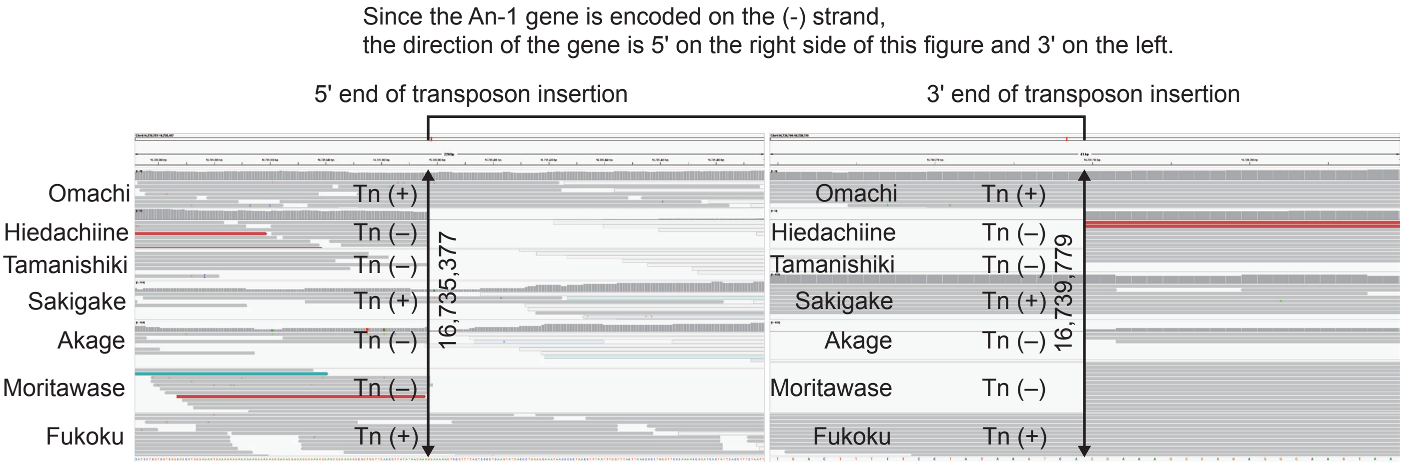


Sup. Fig. 9 Determination of the presence or absence of a transposon in the *An-1* promoter

The alignment of the NGS reads on the *An-1* promoter region (16,739,780 to 16,735,375) was confirmed by IGV software. Since the reference genome contains a 4.4 kb mutator-like transposon at this position, no reads on this region indicate that a variety has no transposon insertion and *An-1* is functional.

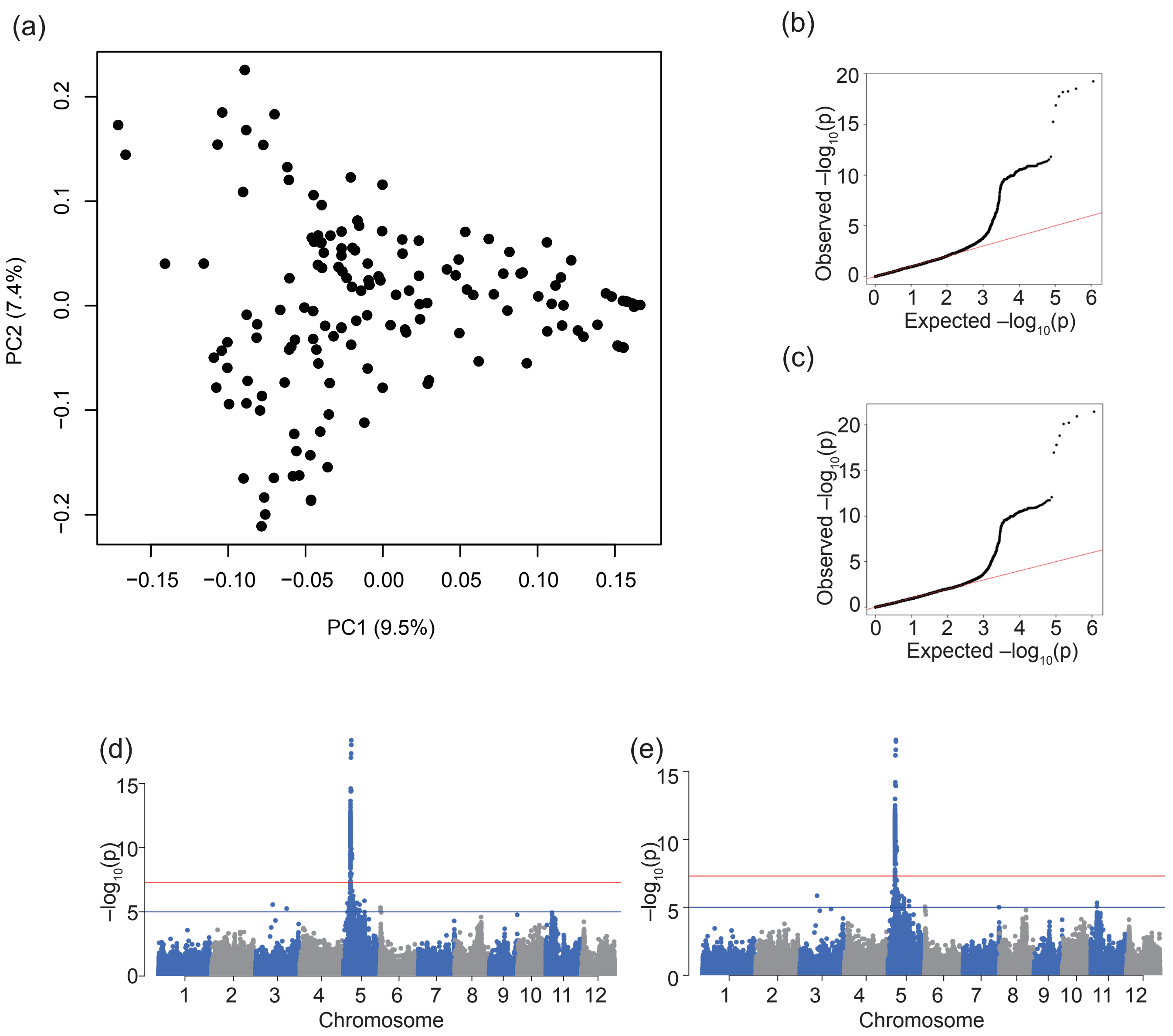


Sup. Fig. 10 PCA analysis and QQ-plots of Figure 6, and GWAS for 128 varieties

PCA for the 153 rice varieties used for GWAS in Figure 6 (a). PC1 and PC2 indicate the scores of principal components 1 and 2, respectively. Values in parentheses indicate the percentage of variance in the data explained by each principal component. (b and c) QQ-plots of GWAS for the degree of awn formation (b), and awn length (c). Since 25 varieties have one year of data (Sup. Table 7 and 8), we excluded these 25 cultivars and performed GWAS on the remaining 128 varieties for the degree of awn formation (d), and awn length (e).

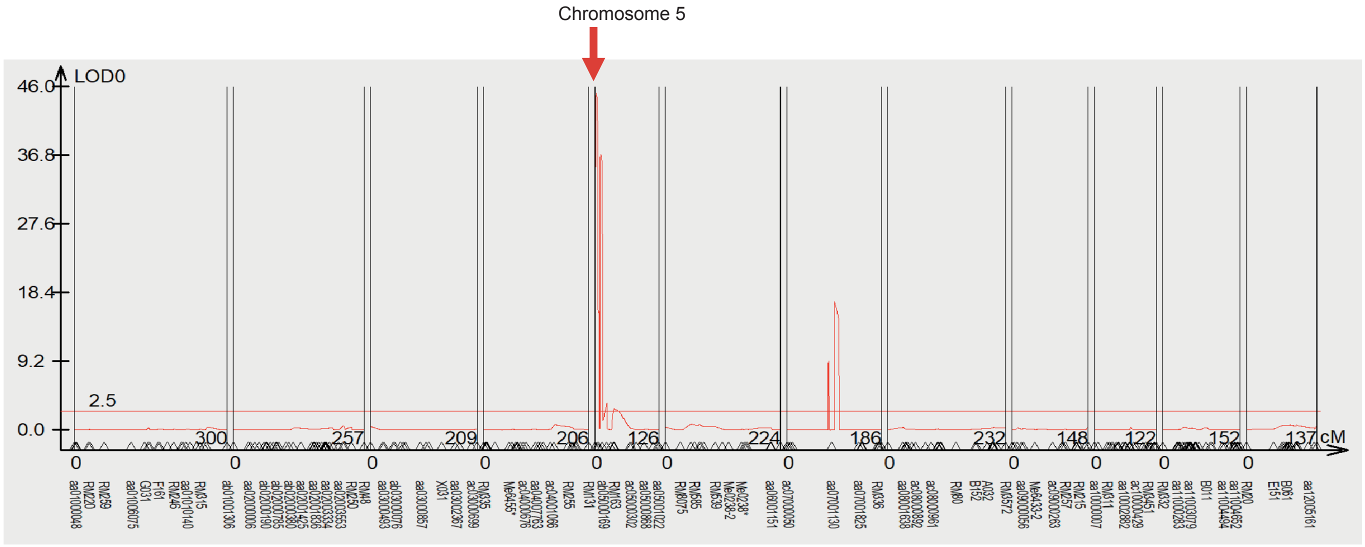


Sup. Fig. 11 QTL analysis for the awn formation with double haploid lines (DHLs) obtained from crosses between “Reiho” and “Yamadanishiki”

The arrow indicates the uppermost QTL.

(a)

**LOC_Os05g10630.1/Os05g0194600** (5791922..5797158 (- strand)): O-sialoglycoprotein endopeptidase

T (5793349, GCA->ACA;A->T)

| | |
|---|---|
| Os05t0194600-01_Osat/1-380 | DVSFSGILSFIEATAIEKLK---NNECTP-----------ADLCYSL-QETLFAMLVEIT |
| BGIOSGA018653-PA_Oind/1-380 | DVSFSGILSFIEATAIEKLK---NNECTP-----------ADLCYSL-QETLFAMLVEIT |
| ORUFI05G06540.1_Oruf/1-394 | DVSFSGILSFIEATAIEKLK---NNECTP-----------ADLCYSL-QETLFAMLVEIT |
| ONIVA05G06750.1_Oniv/1-1086 | DVSFSGILSFIEATAIEKLK---NNECTP-----------ADLCYSL-QETLFAMLVEIT |
| OBART05G06150.1_Obar/1-405 | DVSFSGILSFIEATAIEKLK---NNECTP-----------ADLCYSL-QETLFAMLVEIT |
| ORGLA05G0055200.1_Ogla/1-380 | DVSFSGILSFIEATAIEKLK---NNECTP-----------ADLCYSL-QETLFAMLVEIT |
| OGLUM05G06430.1_Oglu/1-440 | DVSFSGILSFIEATAIEKLK---NNECTP-----------ADLCYSL-QETLFAMLVEIT |
| OMERI05G05650.1_Omer/1-380 | DVSFSGILSFIEATAIEKLK---NNECTP-----------ADLCYSL-QETLFAMLVEIT |
| OPUNC05G05610.2_Opun/1-402 | DVSFSGILSFIEATAIEKLK---NNECTP-----------ADLCYSL-QETLFAMLVEIT |
| OB05G15610.1_Obra/1-379 | DVSFSGILSFIEATAVEKLK---NNECTP-----------ADLCYSL-QETLFAMLVEIT |
| LPERR05G05180.1_Lper/1-442 | DVSFSGILSFIEATAIEKLK---SNECTP-----------ADLCYSL-QETLFAMLVEIT |
| EME29264_Gsul/1-201 | DVSFSGLLSLMEEQLDNWLT---RQGYTV-----------ADLCFSL-QETVFSMLVEVT |
| CDF35306_Ccri/1-343 | DVSFSGLLSFIETEARKALK---DGSFTV-----------EDLCFSL-QETVFAMLVEVT |
| ERN02003_Atri/1-368 | DVSFSGILSYIEATAAEKLQ---NNECTP-----------ADLCFSL-QETVFAMLVEIT |
| scaffold_702083.1_Alyr/1-353 | DVSFSGILSYIETTAEEKLK---NNECTP-----------ADLCYSL-QETVFAMLVEIT |
| OMO52917_Ccap/1-577 | DVSFSGILSYIEATAEEKLK---NNECTP-----------ADLCYSL-QETLFAMLVEIT |
| KZM89242_Dcar/1-244 | DVSFSGILSYIETTAEEKLK---NKECTP-----------ADLCYSL------------- |
| EFJ17849_Smoe/1-337 | DVSFSGILSYIESVATEKLA---AKECTP-----------ADLCFSLQQETVFAMLVEIT |
| OQU77586_Sbic/1-381 | DVSFSGILSFIEATAIEKLK---NNECTP-----------ADLCYSL-QETVFAMLVEIT |
| OMO71358_Ccap/1-353 | DVSFSGILSYIEATAEEKLK---NNECTP-----------ADLCYSL-QETLFAMLVEIT |
| ESW19252_Pvul/1-352 | DVSFSGILSYIEATAVEKLK---NNECTP-----------ADLCYSL-QETLFAMLVEIT |
| EFJ16384_Smoe/1-337 | DVSFSGILSYIESVATEKLA---AKECTP-----------ADLCFSLQQETVFAMLVEIT |
| PNT71658_Bdis/1-381 | DVSFSGILSYIEAAAIEKLK---SNECTP-----------ADLCYSL-QETLFAMLVEIT |
| Tp57577_TGAC_v2_mRNA39089_Tpra/1-352 | DVSFSGILSFIEATANEKLK---NNECTP-----------ADLCYSL-QETLFAMLVEIT |
| PGSC0003DMT400027091_Stub/1-346 | DVSFSGILSFIEATAEEKLK---NNECSP-----------ADLCFSL-QETLFAMLVEIT |
| CDX79172_Bnap/1-423 | DVSFSGILSYIETTAEEKLK---NNECTP-----------ADLCYSL-QETLFAMLVEIT |
| OTF84838_Hann/1-365 | DVSFSGILSYIESEAEQKLK---NNECTP-----------ADLCYSL-QETLFAMLVEIT |
| KQL13880_Sita/1-381 | DVSFSGILSFIEATAIEKLK---NNECTP-----------ADLCYSL-QETLFAMLVEIT |
| Bra013635.1-P_Brap/1-424 | DVSFSGILSYIETTAEEKLK---NNECTP-----------ADLCYSL-QETLFAMLVEIT |
| Bo1g028550.1_Bole/1-353 | DVSFSGILSYIETTAEEKLK---KNECTP-----------ADLCYSL-QETLFAMLVEIT |
| AB099179_Oluc/1-374 | DVNLSGILTFYETYAKEHLG---AGEVTV-----------ADLCMSM-QETVFSMLVEIT |
| KJB27873_Grai/1-354 | DVSFSGILSYIEATAEEKLK---NNECTP-----------ADLCYSL-QETLFAMLVEIT |
| ENSCSAVP00000005802_Csav/1-342 | DISFSGLLSFIEMAANTKIA---SGECTAED-----------LCFSL-QETVFAMLVEIT |
| ONI29401_Pper/1-350 | DVSFSGILSYIEATAVEKLK---NSECTP-----------ADLCYSL-QENVFAMLVEIT |
| KEH43343_Mtru/1-349 | DVSFSGILSYIEATANEQLK---NNECTP-----------ADLCYSL-QETLFAMLVEIT |
| KMT15796_Bvul/1-358 | DVSFSGILSYIETTAEEKLK---NNECTP-----------ADLCYSL-QETLFAMLVEIT |
| OAY51927_Mesc/1-349 | DVSFSGILSFIEATAEEKLK---NNECTP-----------ADLCYSL-QETVFAMLVEIT |
| CDX82960_Bnap/1-353 | DVSFSGILSYIETTAEEKLK---KNECTP-----------ADLCYSL-QETLFAMLVEIT |
| PNS94955_Ptri/1-360 | DVSFSGILSFIEATTEEKLK---NNECTP-----------ADLCYSL-QETVFAMLVEIT |
| PNS22680_Ptri/1-242 | DVSFSGILSFIEATTEEKLK---NNECTP-----------ADLCYSL-QETVFAMLVEIT |
| SapurV1A.1606s0010.1 | MDVSFSGILSFIEATTEEKLK---NNECTP-----------ADLCYSL-QETVFAMLVEIT |
| GSMUA_Achr7P10790_001_Macu/1-347 | DVSFSGILSYIEATAVEKLK---NNECTP-----------ADLCYSL-QETLFAMLVEIT |
| AT4G22720.1_Atha/1-353 | DVSFSGILSYIETTAEEKLK---NNECTP-----------ADLCYSL-QETVFAMLVEIT |
| TraesCS1A02G123600.1_Taes/1-381 | DVSFSGILSFIEAAAIEKLE---NNECTP-----------ADLCYSL-QETLFAMLVEIT |
| GSMUA_Achr10P26500_001_Macu/1-445 | NVSFSGILSYIEATAVEKLK---NNECTP-----------ADLCYSV-QETVCAMLVEII |
| TraesCS1D02G130800.1_Taes/1-381 | DVSFSGILSFIEAAAIEKLE---NNECTP-----------ADLCYSL-QETLFAMLVEIT |
| TraesCS1B02G143400.1_Taes/1-381 | DVSFSGILSFIEAAAIEKLE---NNECTP-----------ADLCYSL-QETLFAMLVEIT |
| HORVU1Hr1G028330.1_Hvul/1-431 | DVSFSGILSFIEAAAIEKLE---NNECTP-----------ADLCYSL-QETLFAMLVEIT |
| Zm00001d037675_P001_Zmay/1-381 | DVSFSGILSFIEAAAIEKLK---NNECTP-----------ADLCYSL-QETIFAMLVEIT |
| TRIDC1AG017720.2_Tdic/1-394 | DVSFSGILSFIEAAAIEKLE---NNECTP-----------ADLCYSL-QETLFAMLVEIT |
| AET1Gv20305500.1_Atau/1-400 | DVSFSGILSFIEAAAIEKLE---NNECTP-----------ADLCYSL-QETLFAMLVEIT |
| TRIDC1BG021880.1_Tdic/1-413 | DVSFSGILSFIEAAAIEKLE---NNECTP-----------ADLCYSL-QETLFAMLVEIT |
| KRH03601_Gmax/1-352 | DVSFSGILSYIEATAAEKLK---NNECTP-----------ADLCYSL-QETLFAMLVEIT |
| KRG97103_Gmax/1-352 | DVSFSGILSYIEATAAEKLK---NNECTP-----------ADLCYSL-QETLFAMLVEIT |
| PSS35852_Achi/1-342 | DVSFSGILSYIEATAEEKLK---NNECTP-----------ADLCYSL-QETLFAMLVEIT |
| Vradi0083s00840.1_Vrad/1-352 | DVSFSGILSYIEATAVEKLK---NNECTP-----------ADLCYSL-QETLFAMLVEIT |
| Solyc02g068750.1.1.1_Slyc/1-346 | DISFSGILSFIEATAEEKLK---NNECSP-----------ADLCFSL-QETLFAMLVEIT |
| PVH61867_Phfi/1-421 | DVSFSGILSFIEATAIEKLK---NNECTP-----------ADLCYSL-QETVFAMLVEIT |
| Pp3c8_19600V3.3_Ppat/1-339 | DVSFSGLLSFVEELAARTLN---DNEITP-----------ADLCFSL-QETVFAMLVEIT |
| PUZ64343_Phha/1-421 | DVSFSGILSFIEATAIEKLK---NNECTP-----------ADLCYSL-QETVFAMLVEIT |
| YKR038C_Scer/1-386 | DLSMSGILASIDLLAKDLFKGNKKNKILFDKTTGEQKVTVEDLCYSL-QENLFAMLVEIT |
| PTQ37885_Mpol/1-370 | DVSFSGIESFIEETANRKLA---ANECTP-----------ADLCFSL-QETVFAMLVEIT |
| Y71H2AM.1.1_Cele/1-337 | DVSLSGILSLIEKKAPKLIE---SGDFTPED-----------LCFSL-QETVFAMLIEIT |
| KVH95372_Ccar/1-384 | DVSFSGILSYIEATAEEKLK---NNDCTP-----------ADLCYSL-QETLFAMLVEIT |
| PHT89813_Cann/1-346 | DVSFSGILSYIEATTEEKLK---SNECTP-----------ADLCFSL-QETLFAMLVEIT |
| CD098614_Ccan/1-147 | DVSFSGILSYIESTAEEKLK---DNECTP-----------ADLCYSL-QETLFAMLVEIT |
| TRITD1Bv1G067590.1_Ttur/1-230 | DVSFSGILSFIEAAAIEKLE---NNECTP-----------ADLCYSL-QETLFAMLVEIT |
| TRITD1Av1G058470.1_Ttur/1-375 | DVSFSGILSFIEAAAIEKLE---NNECTP-----------ADLCYSL-QETLFAMLVEIT |
| itb06g18010.t1_Itri/1-355 | DVSFSGILSYIEATTEEMLK---NNECSP-----------ADLCYSL-QETLFAMLVEIT |
| FBpp0075139_Dmel/1-347 | DVSFSGILSYIEDLAEPGKR--QNKRKKPQE-EEVNNYSQADLCYSL-QETIFAMLVEIT |
| itb09g15640.t1_Itri/1-353 | DVSFSGILSYIEATTEEMLK---NNECSP-----------ADLCYSL-QETLFAMLVEIT |
| Pav_sc0006064.1_g070.1.mk:mrna_Pavi/1-148 | DVSFSGILSYIEATAVEKLK---NSECTP-----------ADLCYSL-QENVFAMLVEIT |
| ESR44632_Ccle/1-357 | DVSFSGILSYIEATAAEKLN---NNECTP-----------ADLCYSL-QETLFAMLVEIT |
| ESR44641_Ccle/1-132 | NVSFSRILSYIEATAAEKLN---NNECTP-----------ANLCYSL-QETLFAMLMEIT |
| CMD081CT_Cmer/1-351 | DVSFSGILSRIQDLIGSYAI--------------------PDLCFSL-QETVFAMLVEVT |
| GBG63216_Cbra/1-346 | DVSFSGLLSYAETTAAELLE---KGNYTP-----------EDLCFSL-QETVFAMLVEIT |
| EOY04854_Tcma/1-351 | DVSFSGILSYIEATAEEKLK---NNECTP-----------ADLCYSL-QETLFAMLVEIT |
| Tc04v2_p014270.2_Tccr/1-351 | DVSFSGILSYIEATAEEKLK---NNECTP-----------ADLCYSL-QETLFAMLVEIT |
| cds-EVM0006908.1_Pver/1-353 | DVSFSGILSYIEATAEEKLK---NNECTP-----------ADLCYSL-QETLFAMLVEIT |
| Oeu061601.1_Oesy/1-271 | DVSFSGILSYIEATAEEKLK---ANECTP-----------ADLCYSL-QETLFAMLVEIT |
| TRIUR3_22527-P1_Tura/1-375 | DVSFSGILSFIEAAAIEKLE---NNECTP-----------ADLCYSL-QETLFAMLVEIT |
| maker-chr09-exonerate_est2genome-gene-43.9-mRNA-1:cds_Cmel/1-195 | DVSFSGILSYIESTAEEKLK---SNECTP-----------ADLCYSL-QETLFAMLVEIT |
| PRQ55908_Rchi/1-359 | DVSFSGILSYIEATAVEKLK---NDECTP-----------ADLCHSL-QNV--------- |
| PRQ56021_Rchi/1-356 | DVSFSGILSYIEATAVEKLK---NDECTP-----------ADLCYSL-QENVFAMLVEIT |
| HORVU.MOREX.r2.1HG0023690.1.mrna1_Hvgo/1-381 | DVSFSGILSFIEAAAIEKLE---NNECTP-----------ADLCYSL-QETLFAMLVEIT |
| TKW25347_Svir/1-381 | DVSFSGILSFIEATAIEKLK---NNECTP-----------ADLCYSL-QETLFAMLVEIT |
| NC6G0039400.1:cds_Ncol/1-436 | DVSFSGILSYIEATATEKLE---KNECTP-----------ADLCFSL-QETLFAMLVEIT |
| Csa10g028240.1_Csat/1-359 | DVSFSGILSYIETTAEEKLK---NNECTP-----------ADLCYSL-QETVFAMLVEIT |
| Cla97C10G192000.1_Clan/1-340 | DVSFSGILSYIEATAEEKLK---SNECTP-----------ADLCYSL-QETLFAMLVEIT |
| cds.novel_model_6425_5bd9a17a_Csfe/1-353 | DVSFSGILSFIEATAEEKLK---NNECTP-----------ADLCYSL-QETLFAMLVEIT |
| Csa12g034140.1_Csat/1-353 | DVSFSGILSYIETTAEEKLK---NNECTP-----------ADLCYSL-QETVFAMLVEIT |
| ENSP00000206542_Hsap/1-335 | DVSFSGILSFIEDVAHRMLA---TGECTPED-----------LCFSL-QETVFAMLVEIT |
| VVA34870_Pdul/1-350 | DVSFSGILSYIEASAVEKLK---NSECTP-----------ADLCYSL-QENVFAMLVEIT |

(b)

LOC_Os05g10650/Os05g0194900 (5813265-5815545):"6-phosphofructokinase, putative, expressed"

```
                                                            G (5815242, CGC->GGC;R->G)
LOC_Os05g10650.1            EEPIVPLVEGENSLV--KAPPLLANAGD----RAALCNGAA*--------------------------------  542
GRMZM2G401970_P01           EEPHMPLVEGENALF--AN-PSMCNG------NGHLCNGHKA*-------------------------------  526
Si021760m                   EEPHLPLVEGENALV--RSPSTMCNG------NGHLCSGAA*--------------------------------  522
Pavir.J04698.1.p            EEPHLPLVEGENALV--VRSPSMCNGNG----NGHLCSGAA*--------------------------------  540
Glyma.04G086900.1.p         EQPPTELLEGNNCNDVGDAGKEEQPPKDEQESGNHADNGIKTEN*-----------------------------  509
AT4G26270.1                 EKPMSALLDDGNCNGVVDVPPVT-----KEITK*----------------------------------------  489
Sobic.009G071800.1.p        EEPHMPLVEGEYAL---VRSPSMCNG------NGHLCSGAA*--------------------------------  536
Pp3c9_24570V3.1.p           VENEKSTLSSN-----GASKPGNERATED--ESVLIHSGDRQEFPVSAGGLQPSPISSPVAGEPTPSVDHAAP*  622
Solyc07g045160.2.1          EEPPTQLSDDATTDDNLMEKQILTF*------------------------------------------------  490
150344                      ESPP-ASIGTV-----GG----EHTATPE--NGIPRRVG--------LSGVTE-----NL-G*-----------  511
```

(c)

LOC_Os05g10670/ Os05g0195200 (5846045-584829):"zinc finger CCCH type family protein, putative, expressed"

```
                                                                       A (5847976, ACG->GCG;T->A)
LOC_Os05g10670.1            HA-----PPWQQHVASPVSGVEGGGGRESEVVAAPYH---LLDTLRHYLPSNEAAAAE-----DEEE--AAAVAAAVDAY      135
GRMZM5G801627_P02           HA-----PPWQR---SPAAS-----GVTDADDASPYA---LLAALQHYLPSNEVA----AYDEDDEEALAAATAAVDAY      69
Si024826m                   HA-----PPWQQ---PPVSG-----GGMDGDDASPYT---LLAALRHYLPSNEAA---AAYDEDDEEA-----LAAVDAY      64
Pavir.J09277.1.p            HA-----PPWQQQQQPAASA-----GMVDGDDASPYS---LLVALRHFLPSNEAAA--AAYDEDDEEA-----LAAVDAY      68
Glyma.08G031400.1.p         HV-----PPWTP-------EIF--SPYTGNADYSPYSLQEALSALQHYESTDAESDSEFP---------SREPEVPVDAY      70
AT4G29190.1                 EI-----PPWPVLEELTTSEFF--SPVMNSPD---CSMLEALAGLQRYLPSNEPDPESYP--------DLLGPDSPIDAY      75
Sobic.009G072100.1.p        YA-----PPWQQSPA----A-----SMDADEAASPYA---LLAALQHYLPSNEVSAAAAPYDEDDEEAALA---AGVDAY      68
Pp3c5_14090V3.1.p           VGKGGGRAELRGLSLAPGSPLGGGGSSMGS-------PTFSDALFNKFLPSNNEDEEA---------------WPAVDMY      204
Solyc07g053750.1.1          -------PPRKLLSRRA--TLSSIDHVVSDVQFMDSPKSESDAQFQKFLPYNSLDDD-----------------DDADPY      83
441947                      SS-----PPSSPVDS----------PRG------TSHDAALSTVLHRFLPSNNSDEAT---------------WSAEDVY      111
                                                                        :.::   :                         * *
```

Sup. Fig. 12 Evaluation of amino acid substitutions in the three candidates genes

Amino acid sequences of the candidates are compared with homologs from other plants. Rice (LOC_Os05g10630, LOC_Os05g10650, LOC_Os05g10670), Maize (Zm00001d037675_P001_Zmay, GRMZ2G401970_PO1, GRMZM5G801627_P02), foxtail millet (KQL13880_Sita, Si021760m, Si024826m), switchgrass (Pav_sc0006064.1_g070.1.mk:mrna_Pavi, Pavir.J04698.1.p, Pavir.J09277.1.p), sorghum(OQU77586_Sbic, Sobic.009G071800.1.p, Sobic.009G072100.1.p), soybean(KRH03601_Gmax, KRG97103_Gmax, Glyma.04G086900.1.p, Glyma.08G031400.1.p), Arabidopsis(AT4G22720.1_Atha, AT4G26270.1, AT4G29190.1), tomato (Solyc02g068750.1.1.1_Slyc, Solyc07g045160.2.1, Solyc07g053750.1.1), *Selaginella moellendorffii* (EFJ16384, 150344, 441947)and *Physcomitrium patens* (Pp3c8_19600V3.3_Ppat, Pp3c9_24570V3.1.p, Pp3c5_14090V3.1.p). The amino acid substitutions in the two candidate genes (LOC_Os05g10650 and LOC_Os05g10670) were found to be unlikely to affect their function in less conserved regions, whereas the substitutions in two others are located in well conserved regions. We then investigated these two candidates in more detail.

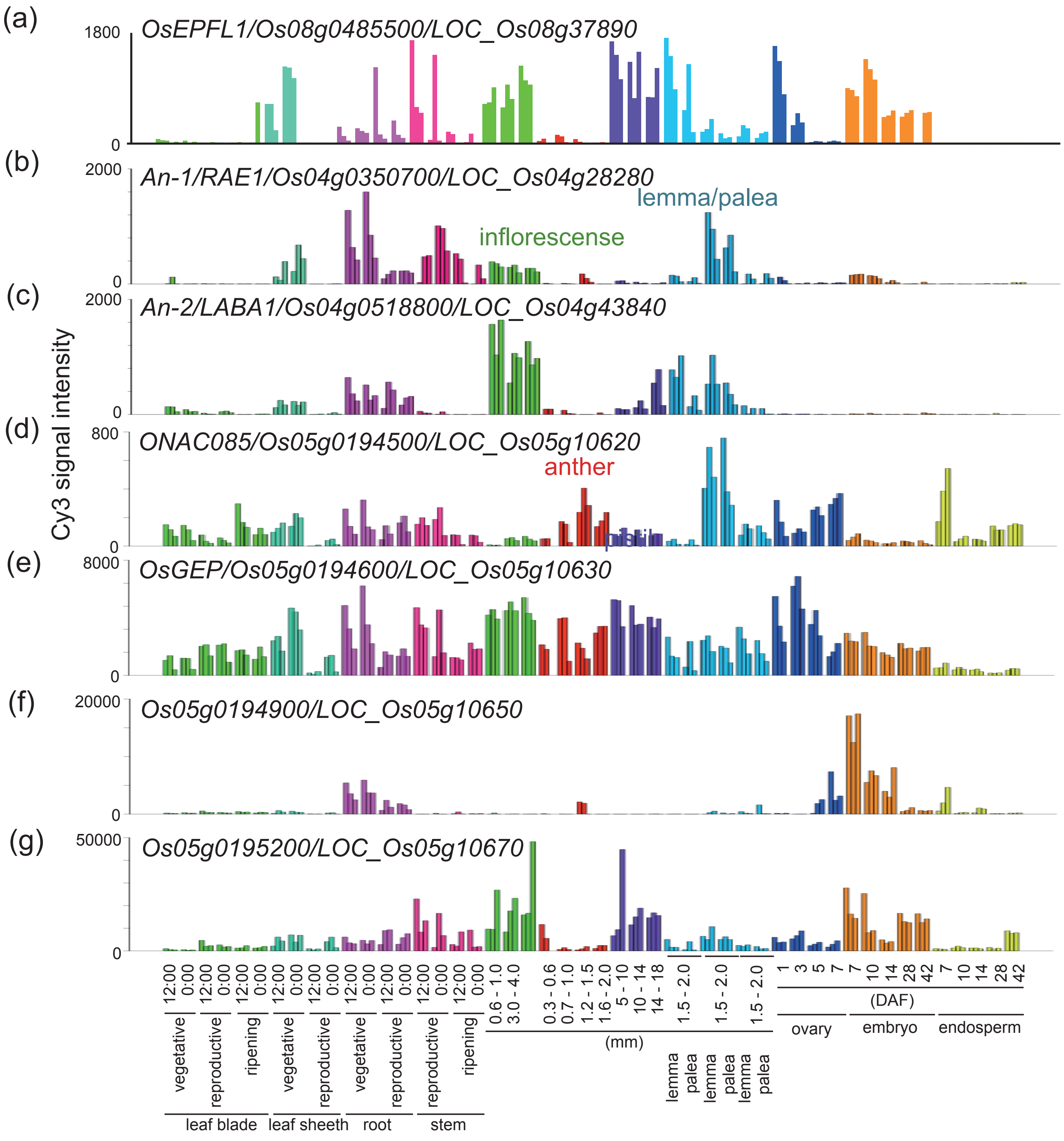


Sup. Fig. 13 Public expression data for the already known awn forming genes and the four candidate genes

The expression of *OsEPFL1* (a and h), *An-1* (b and i), *An-2* (c and j), *ONAC085* (d and k), *OsGEP* (e and l), *Os05g0194900/LOC_Os05g10650* (f and m), *Os05g0195200/LOC_Os05g10670* (g and n). Temporal and spatial expression pattern (a-g) was obtained from RiceXpro (https://ricexpro.dna.affrc.go.jp/), while the expression under abiotic stress condition (h-n) was obtained from TENOR (Transcriptome ENcyclopedia Of Rice) database (https://tenor.dna.affrc.go.jp/). DAF, days after flowering; ABA, abscisic acid; JA, jasmonic acid.

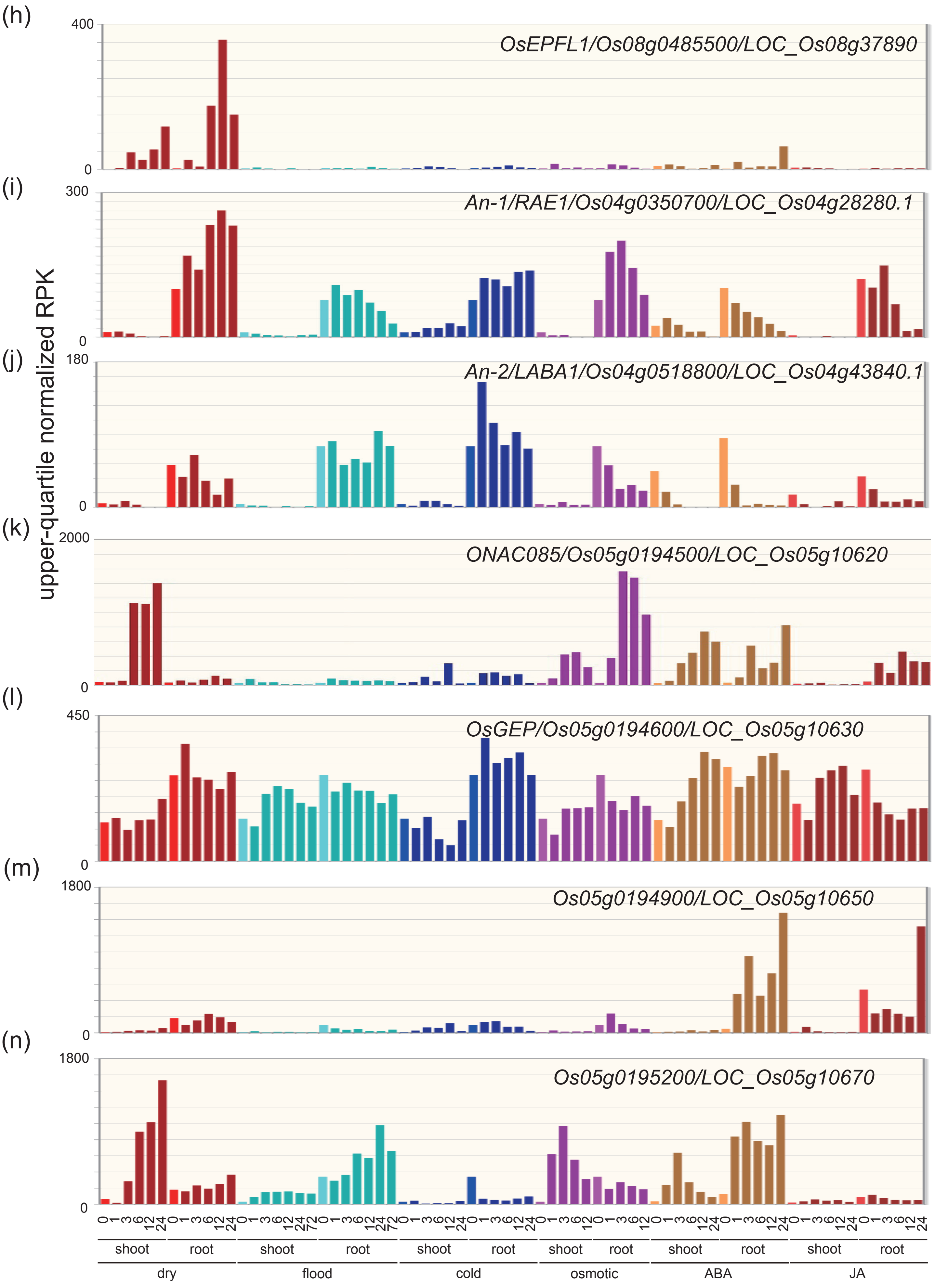


Supplemental Figure 13 Suganami et al. continued

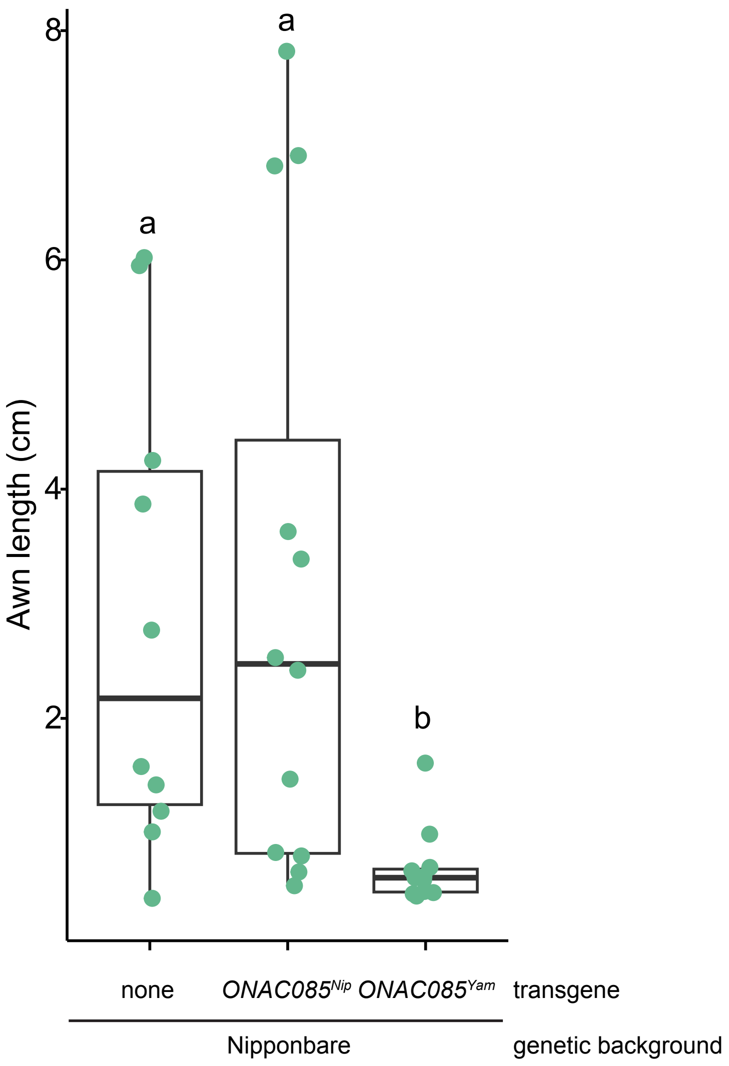


Sup Fig 14. Awn length of the transgenic and control plants shown in Fig. 7a, c, e, f

n > 10. Tukey' s HSD test was applied for multiple comparisons; different letters show significance at 5% level.

(a)

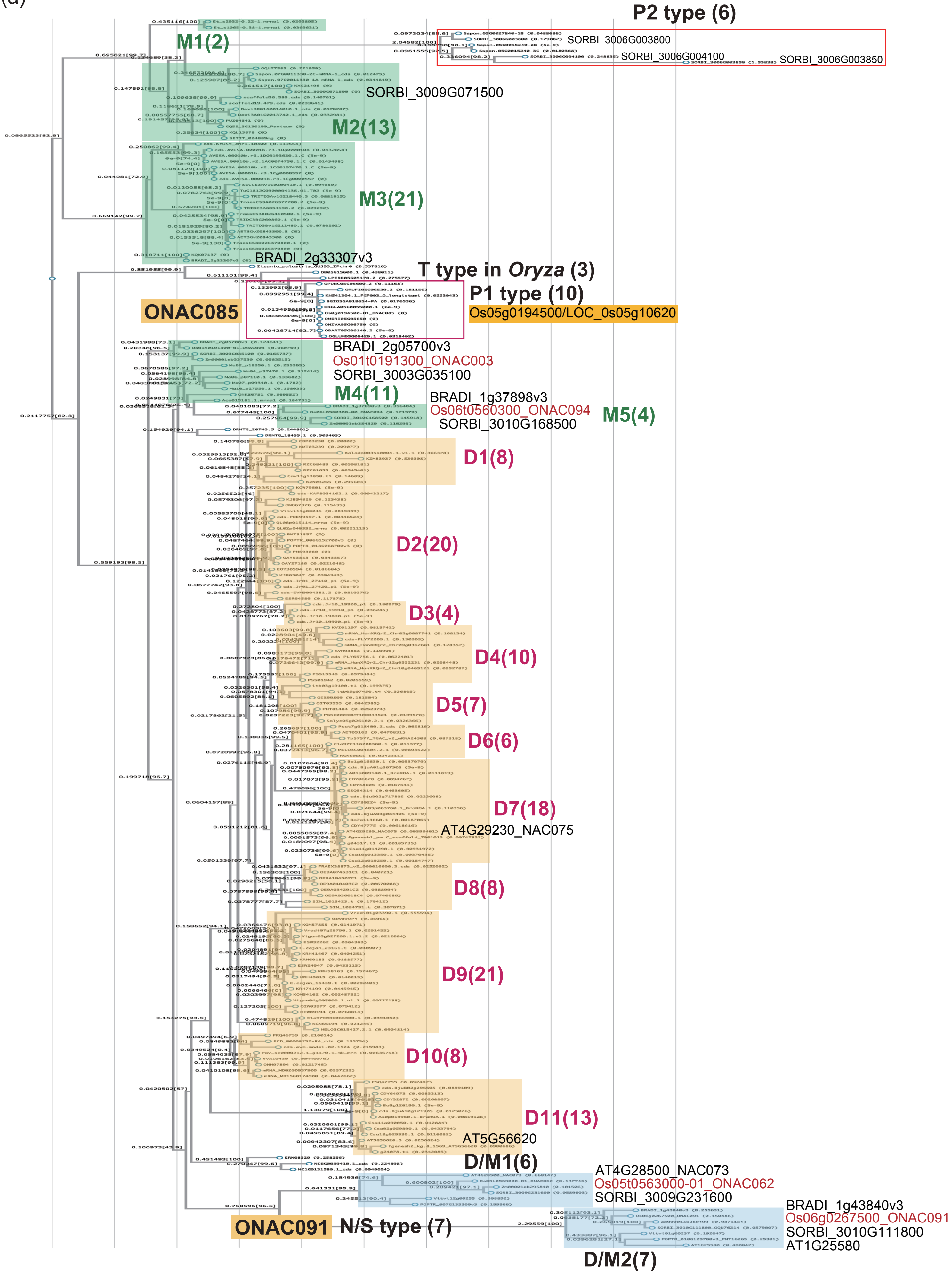


Supplemental Figure 15 Suganami et al.

(b)

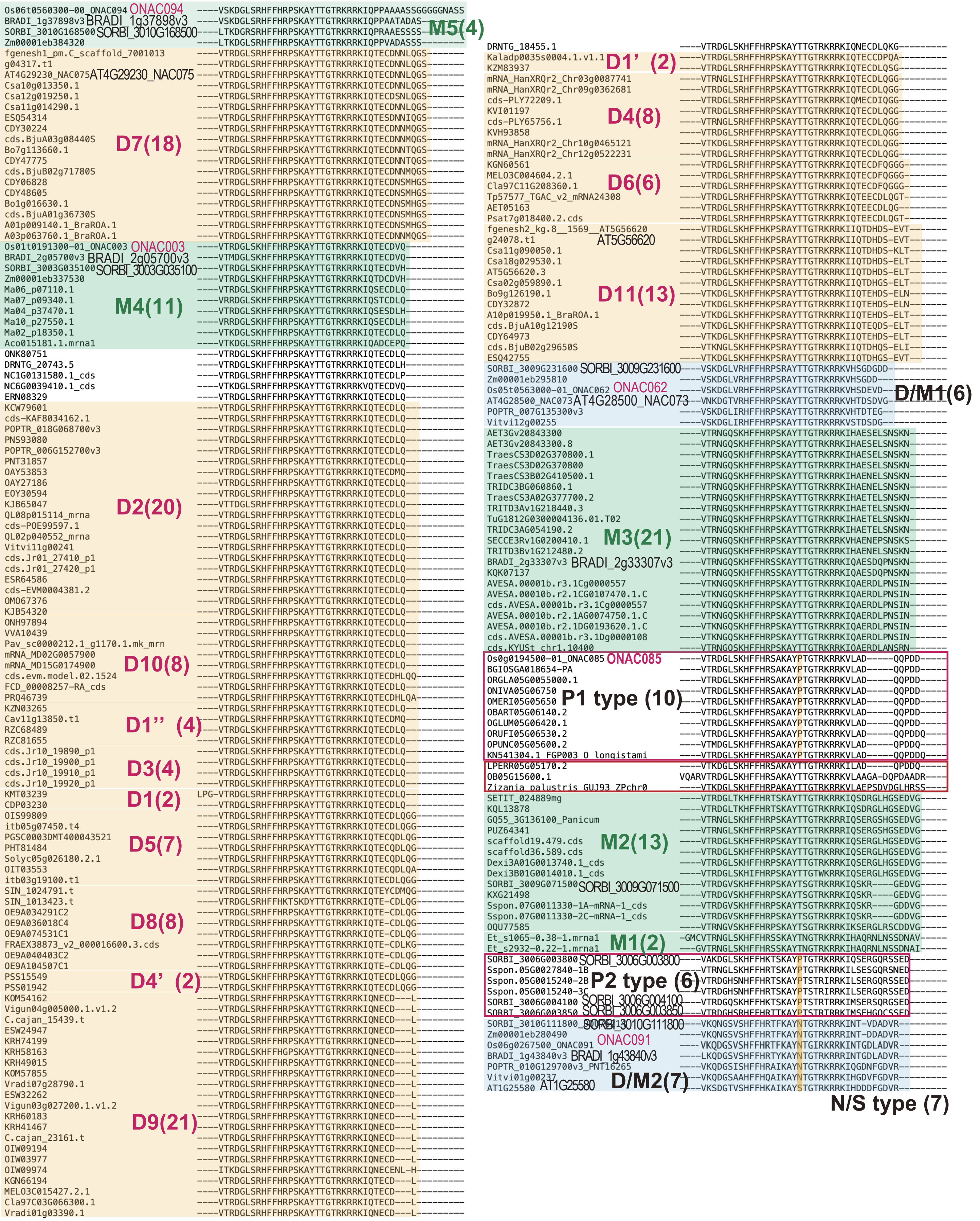

Sup. Fig. 15 Phylogenetic analysis of ONAC085.

A phylogenetic tree was constructed by comparing a wide collection of available plant sequences for homologous genes of ONAC085 (a). Amino acids in the regions surrounding the mutations found in this study were compared (b).
